# Genome-wide associations spanning 194 in-hospital drug dosage change phenotypes highlight diverse genetic backgrounds in concurrent drug therapy

**DOI:** 10.1016/j.csbj.2025.06.042

**Published:** 2025-06-25

**Authors:** Alexander Pil Henriksen, Cristina Leal Rodríguez, Hannah Currant, Ioannis Louloudis, Jorge Hernansanz Biel, Maria Herrero-Zazo, Ewan Birney, Thomas Folkmann Hansen, Gianluca Mazzoni, Amalie Dahl Haue, Henning Bundgaard, Christian Erikstrup, Khoa Manh Dinh, Liam Quinn, Mie Topholm Bruun, Henrik Hjalgrim, Erik Sørensen, Christina Mikkelsen, Michael Schwinn, Ole Birger Vestager Pedersen, Henrik Ullum, Sisse Rye Ostrowski, DBDS Genomic Consortium, Karina Banasik, Søren Brunak

**Affiliations:** a Novo Nordisk Foundation Center for Protein Research, Faculty of Health and Medical Sciences, University of Copenhagen, Copenhagen, Denmark; b Copenhagen Prospective Studies on Asthma in Childhood, Copenhagen University Hospital, Gentofte, Denmark; c Nuffield Department for Population Health, University of Oxford, Oxford, UK; d European Molecular Biology Laboratory, European Bioinformatics Institute, Hinxton, UK; e Danish Headache Center, Department of Neurology, Copenhagen University Hospital, Rigshospitalet-Glostrup, Copenhagen, Denmark; f The Heart Center, Rigshospitalet, Copenhagen University Hospital, Copenhagen, Denmark; g Department of Clinical Medicine, Faculty of Health and Medical Sciences, University of Copenhagen, Copenhagen, Denmark; h Department of Clinical Immunology, Aarhus University Hospital, Aarhus, Denmark; i Department of Clinical Medicine, Health, Aarhus University, Aarhus, Denmark; j Department of Clinical Immunology, Copenhagen University Hospital - Rigshospitalet, Copenhagen, Denmark; k Department of Clinical Immunology, Zealand University Hospital, Køge, Denmark; l Department of Clinical Immunology, Odense University Hospital, Odense, Denmark; m Danish Cancer Society Research Center, Copenhagen, Denmark; n Department of Epidemiology Research, Statens Serum Institut, Copenhagen, Denmark; o Statens Serum Institut, Copenhagen, Denmark; p Department of Gynecology and Obstetrics, Copenhagen University Hospital Hvidovre, Copenhagen, Denmark

**Keywords:** Pharmacogenomics, Drug-drug interactions, Polypharmacy, Drug dosage

## Abstract

As populations get older and medicine consumption rises, the rate of concurrent drug use and polypharmacy among patients is increasing. Polypharmacy is known to complicate therapy and increase the risk of drug-drug interactions, the individuality of which remain largely unexplored. Here, we perform a series of genome-wide association studies to identify variants associated with dosage changes during episodes of concurrent drug therapy. We extracted in-hospital drug prescription records from 847,537 patients in a population-wide Danish hospital cohort. Using imputed genotype data from the Copenhagen Hospital Biobank and the Danish Blood Donor Study we then performed a series of genome-wide association analyses across 194 drug pair phenotypes fulfilling selection criteria. We identified 51 genome-wide significant (p < 5E-08) loci, 49 so far unreported in any genome-wide association studies, associated with dosage changes across 42 different drug pair phenotypes. 49 of the identified loci were unique to the respective drug pairs. Through annotation of the identified loci, expression quantitative trait loci analyses, and gene-based tests we found links to 57 distinct genes, several of which have previously been associated with disease. This study identifies genes that may modulate response to drug therapy in the context of polypharmacy. Our findings reveal distinct patterns of genetic variation across different drug pairs, suggesting a diverse set of genes involved in drug efficacy and drug response. This study may give a better understanding of the individuality of such mechanisms and may aid the development personalized treatment approaches.

## Highlights

• Drug dosage changes are common among hospital admitted patients.
• We performed GWAS of 194 in-hospital drug dosage change phenotypes.
• Dosage changes show distinct genetic backgrounds across different drug pairs.
• 49 of 51 variants passing genome-wide significance had not been reported in any other GWAS.
• *SEPTIN9*, previously linked to hypertension was here associated with dosage changes in clopidogrel during metoprolol treatment.

## Graphical Abstract

**Figure:**
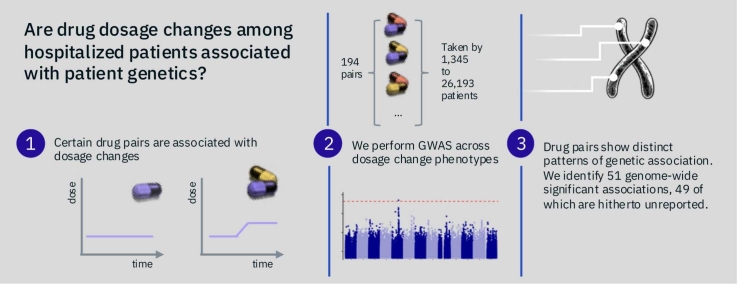

## 1 Introduction

Globally medication consumption is on the rise [1], [2]. There is a notable increase in patients receiving several medications concurrently, particularly among the elderly and others with multimorbidities [3], [4], [5], [6]. It is well established that polypharmacy, commonly defined as taking five or more medications, is associated with adverse drug events (ADEs) as well as higher healthcare costs [7], [8], [9]. Use of multiple drugs may also lead to more subtle therapeutic changes such as changes in drug dosage. In a Danish cohort of more than a million patients Leal et al. recently identified 3993 unique drug pairs, many with known drug-drug interactions (DDIs), which were significantly associated with an increased probability of dosage adjustments [10]. DDIs are well known to be enriched in certain types of drug therapy and have also been shown to contribute to increased rates of hospital admissions, prolonged hospital stays, and more specific clinical outcomes such as bleeding and toxicity [9], [11], [12], [13].

The importance of pharmacogenetics, the effect of individual genetic variation on drug response, is becoming increasingly clear [14]. For example, deleterious variants in drug-metabolizing genes may lead to drug concentrations far outside the therapeutic range. Such drug-gene interactions (DGIs) have been shown to be relevant for up to 24 % of patients receiving drug therapy and the added effects of drug-drug-gene interactions from concurrent medications may raise this level even further [15], [16].

Despite the wealth of documented drug-drug and drug-gene interactions, the underlying biological mechanisms for many interactions remain elusive. Existing databases such as DrugBank, NSIDES, and PharmGKB offer valuable insights but lack full coverage, leaving a substantial portion of DGIs unexplored, especially in the context of polypharmacy [17], [18], [19]. While databases may list hundreds or thousands of DGIs and DDIs, many interactions are without known biological mechanisms and without clinical evidence.

This study aims to address this gap by investigating the genetic underpinnings of dosage adjustments resulting from concurrent drug use. While some dosage changes can reflect standard clinical practice, others may reflect changes in drug absorption, distribution, metabolism, or excretion, processes which may be influenced by interacting drugs and/or genetic factors. In a population of 133,467 genotyped Danish patients covering 436,367 admissions between 2009 and 2016, we perform genome-wide association study (GWAS) analyses to investigate if genetic variants are associated with drug dosage changes in concurrent drug use. We present GWAS results of concurrent drug use phenotypes for 194 drug pairs. The results highlight several genes that may help explain both intended and unintended effects of concurrent drug use.

## 2 Methods

### 2.1 Data sources

In brief, in-patient drug administration data were gathered from electronic healthcare records of more than one million patients admitted to hospitals in Eastern Denmark between 2008 and 2016 (Figure S1). We used medication data covering 185 million treatment episodes described in detail in Leal et al. [10]. These data were organized into drug pair treatment episodes in which individual patients are prescribed two or more drugs at the same time. Each treatment episode included an index drug and a codrug and the daily dose of the index drug was tracked during periods of concurrent prescription as well as during monotherapy (Appendix S1). The analysis identified 3993 drug pairs for which taking the pair of drugs concurrently significantly increased the probability of dosage changes of the index drug when compared to taking the index drug as monotherapy. These pairs are, as in Leal et al. [10], referred to as “dosage adjusted drug pairs”.

Genotype data was retrieved from the Copenhagen Hospital Biobank (CHB) [20], a research biobank which contains samples obtained during workup on hospitalized patients and outpatients at hospitals in the Capital Region of Denmark [20] and from the Danish Blood Donor Study, a large prospective cohort of blood donors initiated in 2010 [21]. Our analyses covered patients from either CHB or DBDS which were included in the Oral Cardio-Metabolic Health Study (CHB-OCMS), a study initiated in 2023 and involving a targeted selection of patients over 18 years of age with cardiometabolic diseases. The CHB-OCMS cohort contains 375,217 patients of which 177,622 had hospital drug records containing any of the 3993 dosage adjusted drug pairs. Genotyping was performed at deCODE genetics using the Illumina Infinium Global Screening Array. Standard quality control measures were applied and imputation was performed using a North-Western European reference as described elsewhere [22]. All genotype data was aligned to the GRCh38 Human Reference Genome build.

For a follow-up analysis, primary care prescription records were retrieved from the Danish National Prescription Registry (DNPR), which contains all prescription drugs collected at Danish community pharmacies [23]. DNPR contains individual-level records for prescriptions redeemed since 1994 and includes information such as date of redemption, drug, brand, dose, and pack size.

### 2.2 Drug pair selection

177,622 patients had hospital drug records containing any of the 3993 dosage adjusted drug pairs as well as accessible genotype information (Table S1). We limited our analysis to the most densely populated, well-mixed population within the overall dataset using visual inspection in the principal component PC1-PC2 space; a population which largely aligns with those who identify as Danish / Northern European. This left 133,467 patients, covering 436,367 separate admissions and 3950,941 treatment episodes. To reduce the number of drug pairs, our analysis was limited to those episodes in which a patient had received more than a single prescription of the index drug. We further excluded drug pairs that included Ibuprofen or Paracetamol as these medications are readily prescribed in hospitals and are often given as “pro re nata” (i.e. by the patient’s needs), making it difficult to know to what extent the patient has taken the drug. When patients had been prescribed a drug pair during multiple separate hospital admissions, we limited our analysis to the first admission to avoid confounding from later clinical intervention. Still, each patient could contribute data to multiple drug pairs. These exclusions left us with 3714 drug pairs. Finally, we used the CaTS Power Calculator to filter drug pairs to only include those which we had a sufficient sample size to detect a genotype relative risk of 1.2 with 80 % power [24]. This left us with 194 drug pairs covering 332,904 admissions and 117,814 patients (Figure S2, Table S2, Table S3). The included drug pairs were prescribed to between 1345 and 26,193 patients.

### 2.3 Genetic analyses

The drug pair selection left us with 194 drug pair phenotypes for which we tested genetic association with dosage changes of the index drug. We encoded dosage change as a binary phenotype with “Change” being when a patient had received a pair of drugs during a hospital admission and the dosage of the index drug had changed between two prescriptions. “Change” covered both instances of dosage increase as well as dosage decrease. “No change” controls had also received the pair of drugs but had not changed dosage of the index drug between two prescriptions. The sample size of the case population (i.e. patients experiencing a “Change”) ranged from 998 to 18,873 across drug pair phenotypes (Table S3).

We performed GWAS for each of the drug pair phenotypes using an additive logistic model with REGENIE v. 3.1 [25]. Model covariates included, sex, year of birth, age at first administration of drug pair, age squared, hospital of admission, genotyping chip, and the first six genetic PCs. The number of PCs was chosen by including the top PCs until the explained variance plateaued (Figure S3). After each GWAS, we removed variants with INFO scores lower than 0.9, multiallelic variants, and variants which did not pass basic quality control in the overall cohort (Minor Allele Frequency > 0.01, genotype missingness < 0.1, Hardy-Weinberg equilibrium exact test p-value < 10^−15^).

GCTA Conditional and Joint Analysis (COJO) as provided through GCTA [26], [27] was used to select significantly associated independent loci which were more than 10 Mb apart. Significant variants were annotated using ANNOVAR [28] and OpenTargets [29]. Where inconsistencies were observed we kept the OpenTargets annotation. Linkage disequilibrium scoring was done using LDSC [30] and gene-based testing was done using fastBAT [31] through GCTA [27]. We tested variant effects on dosage change directionality using linear regression in R v.4.3.3 with all the same covariates as used in the original GWASs. We used the eQTL Catalogue API [32] to perform eQTL mapping. Additionally, FUMA was used to perform gene set enrichment analysis, by comparing with annotated gene sets from KEGG, Reactome and other sources. FUMA’s GENE2FUNC pipeline was run using default settings, submitting only genes identified by location, gene-based testing or eQTLs for each drug pair phenotype. PheWAS results for individual variants were extracted from the OpenTargets database [29]. We corrected for multiple testing using the Bonferroni method counting the number of datasets included (p < 1.25E-05).

### 2.4 Correlation between drug pair phenotype genetic effects

Among the 194 drug pair phenotypes tested for genetic associations many shared index- or codrugs. We speculated whether the genetic background driving dosage change in one drug pair could be similar to that of another drug pair with the same index drug. To calculate the genetic correlation between drug pair phenotypes we created a variant set consisting of all variants that had a p-value of 5E-07 or lower in any of the 194 performed GWASs. This threshold was chosen as only few variants for each drug pair phenotype had p-values smaller than the genome-wide significance threshold of 5E-08 (75 as compared to 974 for the 5E-07 threshold). The effect sizes of the variants in this variant set were then compared and a Pearson correlation was calculated between each combination of two drug pair phenotypes.

### 2.5 Variant effects on primary care prescription patterns

We extracted prescription records from the DNPR (1994–2022) for the 375,217 individuals in the CHB-OCMS cohort. 374,406 (99 %) individuals had available records in DNPR. For each variant of interest, patients were stratified by genotype (0, 1, 2) and prescription records for the two drugs relating to that variant was extracted. Patients included in the GWAS analyses originally identifying each of the 51 genome-wide significant variants were excluded from the analysis. We separated prescription records into treatment episodes where patients had either 1) received the index drug from a drug pair or 2) received both the index drug and codrug in a drug pair within 30 days of each other. We performed linear regression to assess associations between variant genotype and two phenotypes relating to the index drug: 1) number of redeemed prescriptions, and 2) mean dose of redeemed drug. We included sex, date of birth, and age at first prescription as covariates. For both phenotypes we excluded the 1 % most extreme values (1 % top values). We performed all statistical analyses using R v4.3.3.

## 3 Results

### 3.1 Discovery cohort demographics

**Fig. 1:**
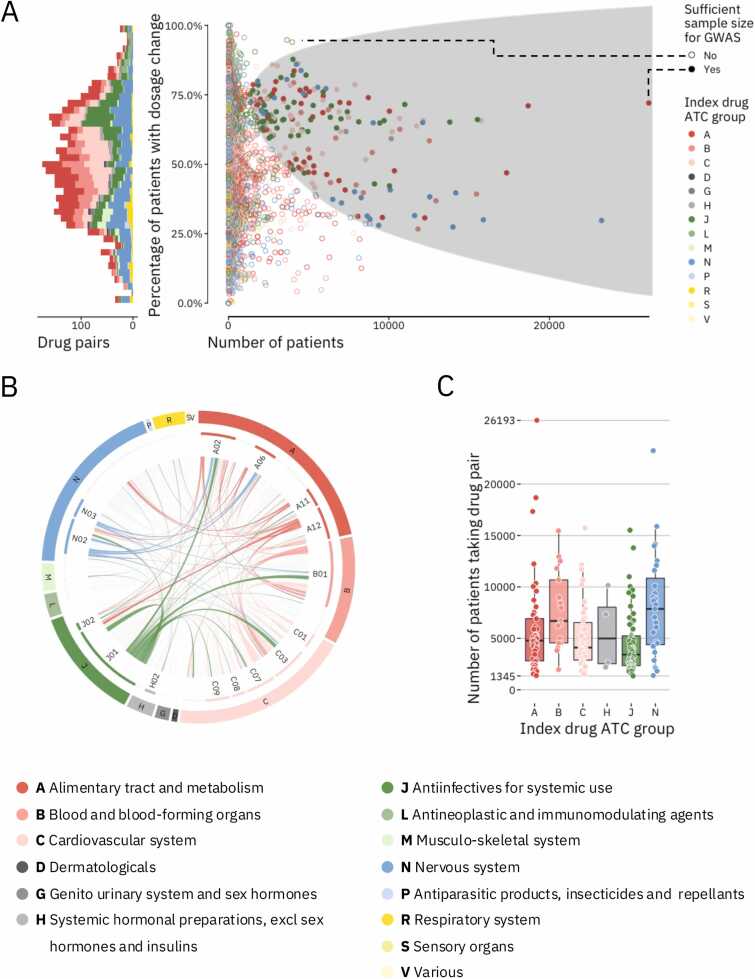
Overview of the selected drug pair phenotypes. (A) Patient counts, and dosage change rates for 3714 relevant drug pairs. Left: A stacked histogram of the drug pairs color-coded by the ATC code of their index drug. Right: Scatter plot showing the number of patients and dosage change rate of each included drug pair. Solid circles are drug pairs administered to enough patients to be selected for GWAS. (B) Circos plot of 3714 relevant drug pairs stratified by ATC drug groups. Links connect the index drug and codrug for each drug pair. Drug pairs with sufficient sample size for GWAS are colored. Links are colored as in A. (C) Number of patients with the selected drug pair phenotypes. Each dot shows the number of patients receiving that drug pair. Boxplots are split by index drug and colored as in A.

We started with 133,467 patients who had been prescribed one of 3993 dosage adjusted drug pairs during hospital admission. The index drugs prescribed to most patients were those belonging to anatomical therapeutic chemical (ATC) classification groups N02 (Analgesics, 53.1 %), J01 (Antibacterials for systemic use, 51.7 %), and B01 (Antithrombotic agents, 45.2 %). The most common index drugs were potassium chloride (a common salt, 40.7 % of admissions), morphine (an analgesic, 38.2 % of admissions), and cefuroxime (an antibiotic, 34.5 % of admissions). We selected 194 drug pairs with sufficient sample size to run a GWAS (Fig. 1A). These drug pairs had been prescribed to between 1345 and 26,193 patients in total with the fraction of patients experiencing a dosage change (cases) varying between 26.8 % and 87.7 %. Of the selected 194 pairs 60.8 % (118) contained drugs from different ATC groups while the pairs with drugs from the same ATC groups were mainly from groups B01 and J01 (Fig. 1B). The median number of patients for the 194 drug pairs was 4529 (sd = 4114), with 59 % of pairs (114) being prescribed to more than 4000 patients (Fig. 1C). Drug pairs with index drugs in ATC group J (Antiinfectives for systemic use) had generally been prescribed to fewest patients (median = 3388 patients) while those with index drugs in ATC group N (Nervous system) had generally been prescribed to most patients (median = 7860 patients).

### 3.2 GWASs identify 51 variants associated with dosage changes

**Fig. 2:**
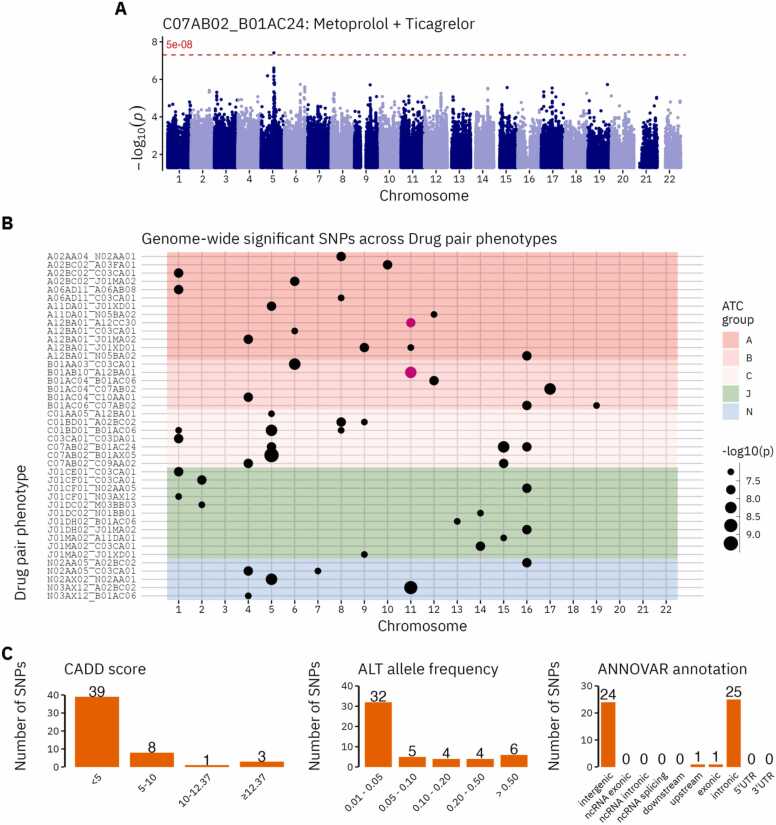
Significant (p < 5E-08) SNP results from the 194 drug pair phenotype GWASs (42 with significant hits). (A). Manhattan plot of the Metoprolol + Ticagrelor drug pair with one significant locus. (B). Discrete scatterplot of all significantly associated SNPs for each drug pair phenotype. The dot size indicates the significance level. Purple dots in the same column indicate pairs of SNPs that are within 1 mbp of each other. The background color indicates the ATC group of the index drug in the drug pair. (C). Distribution of SNPs across CADD scores, allele frequencies and ANNOVAR functional annotations. CADD scores predict the pathogenicity of a SNP with values above 12.37 being considered potentially pathogenic. ANNOVAR annotation categories identify the position of variants relative to nearby genes and their associated function.

We identified 51 genome-wide significant (p < 5E-08) loci associated with dosage changes in 42 of the drug pairs (Fig. 2, Table 1, for full variant information see Table S4. Manhattan- and QQ plots are available in Figure S6). No variants reached the significance level required after multiple test correction with Bonferroni (p < 5E-08 / 194). There was no overlap in the lead variants between drug pair phenotypes. However, rs2511771 (tinzaparin + potassium chloride) and rs56255127 (potassium chloride + magnesium) were located within 1 mbp of each other and both mapped to the *NTM* gene (OpenTargets annotation). Across the 42 GWASs most showed little evidence of genomic inflation with lambda GC values between 0.9927–1.0802 (mean: 1.0302, SE: 0.0032) and LD-score regression intercepts between 0.9768–1.09 (SE: 0.0062–0.0084) (Table S5).

**Table 1:** Genome-wide significant (p < 5E-08) variants identified through the GWAS of 194 drug pair phenotypes. Bold letters in columns 1, and 3 indicate the main ATC group of each drug.

	Index drug		Codrug	rs ID	CHR	p-value	MAF	Closest Gene	Variant ann.
**A**	mag. hydroxide	**N**	morphine	rs117944645	8	2,98E−08	0.010	LRRCC1	intronic
**A**	pantoprazole	**A**	metoclopramide	rs147504573	10	1,09E−08	0.019	KCNMA1	intronic
**A**	pantoprazole	**C**	furosemide	rs116091351	1	1,23E−08	0.017	TMEM81	intronic
**A**	pantoprazole	**J**	ciprofloxacin	rs117452099	6	2,40E−08	0.019	THBS2	intergenic
**A**	lactulose	**A**	sodium picosulfate	rs12736144	1	1,48E−08	0.034	AJAP1	intronic
**A**	lactulose	**C**	furosemide	rs1871838	8	5,43E−08	0.056	DLC1	intergenic
**A**	thiamine	**J**	metronidazole	rs114942430	5	2,85E−08	0.053	CDH6	intergenic
**A**	thiamine	**N**	chlordiazepoxide	rs186107005	12	4,75E−08	0.015	ALG10	intergenic
**A**	potassium chl.	**A**	magnesium	rs56255127	11	2,06E−08	0.135	NTM	intronic
**A**	potassium chl.	**C**	furosemide	rs146985296	6	3,85E−08	0.015	MCM3	intergenic
**A**	potassium chl.	**J**	ciprofloxacin	rs116132368	4	1,50E−08	0.013	UGT2A3	intergenic
**A**	potassium chl.	**J**	metronidazole	rs4757645	11	4,85E−08	0.622	LDHA	intergenic
**A**	potassium chl.	**J**	metronidazole	rs79970770	9	1,10E−08	0.016	ASTN2	intronic
**A**	potassium chl.	**N**	chlordiazepoxide	rs573836037	16	1,09E−08	0.014	HNRNPA1L3	intergenic
**B**	warfarin	**C**	furosemide	NA	6	8,19E−09	0.017	NA	intergenic
**B**	tinzaparin	**A**	potassium chl.	rs2511771	11	7,29E−09	0.661	NTM	intergenic
**B**	clopidogrel	**B**	acetylsal. acid	rs149039924	12	1,04E−08	0.011	CEP83	intronic
**B**	clopidogrel	**C**	metoprolol	rs312802	17	5,27E−09	0.149	SEPTIN9	intronic
**B**	clopidogrel	**C**	simvastatin	rs28636409	4	2,20E−08	0.014	THEGL	intronic
**B**	acetylsal. acid	**C**	metoprolol	rs77925157	16	1,72E−08	0.011	GOT2	intergenic
**B**	acetylsal. acid	**C**	metoprolol	rs758010917	19	3,85E−08	0.059	ZNF331	intronic
**C**	digoxin	**A**	potassium chl.	rs145706366	5	4,13E−08	0.022	CDH18	intronic
**C**	amiodarone	**A**	pantoprazole	rs146704861	8	1,38E−08	0.011	MFHAS1	intergenic
**C**	amiodarone	**A**	pantoprazole	rs370304464	9	4,01E−08	0.159	TLE4	intergenic
**C**	amiodarone	**B**	acetylsal. acid	rs185619351	1	5,36E−08	0.012	IGSF3	intronic
**C**	amiodarone	**B**	acetylsal. acid	rs138489230	5	5,33E−09	0.030	C5orf47	intergenic
**C**	amiodarone	**B**	acetylsal. acid	rs4737862	8	5,79E−08	0.762	PREX2	intergenic
**C**	furosemide	**C**	spironolactone	NA	1	1,12E−08	0.058	MAGI3	intergenic
**C**	metoprolol	**B**	ticagrelor	rs118142978	15	4,16E−09	0.017	GABRA5	intronic
**C**	metoprolol	**B**	ticagrelor	rs77458025	16	1,40E−08	0.026	ERCC4	intergenic
**C**	metoprolol	**B**	ticagrelor	rs111423393	5	1,53E−08	0.050	SLCO4C1	intergenic
**C**	metoprolol	**B**	fondaparinux	rs984113	5	5,46E−10	0.629	MAP3K1	intergenic
**C**	metoprolol	**C**	enalapril	rs151291375	15	1,33E−08	0.026	CDIN1	intergenic
**C**	metoprolol	**C**	enalapril	rs6532537	4	1,46E−08	0.457	UNC5C	intronic
**C**	benzylpenicillin	**C**	furosemide	rs1206902225	1	1,84E−08	0.014	YTHDF2	intronic
**J**	dicloxacillin	**C**	furosemide	rs4668287	2	1,17E−08	0.485	SP5	intronic
**J**	dicloxacillin	**N**	oxycodone	rs111738111	16	1,13E−08	0.016	SLC38A8	intronic
**J**	dicloxacillin	**N**	gabapentin	rs7524537	1	6,73E−08	0.223	DDR2	intronic
**J**	cefuroxime	**M**	chlorzoxazone	rs4675152	2	4,41E−08	0.750	COL4A3	intronic
**J**	cefuroxime	**C**	bupivacaine	rs117134236	14	3,94E−08	0.034	LGMN	intronic
**J**	meropenem	**B**	acetylsal. acid	rs517984	13	5,33E−08	0.284	OXGR1	intronic
**J**	meropenem	**J**	ciprofloxacin	rs76796131	16	2,42E−08	0.047	C16orf78	intronic
**J**	ciprofloxacin	**A**	thiamine	rs73370371	15	4,00E−08	0.027	OCA2	intronic
**J**	ciprofloxacin	**C**	furosemide	rs55731481	14	2,04E−08	0.018	BCL11B	intergenic
**J**	ciprofloxacin	**J**	metronidazole	rs2618023	9	3,46E−08	0.022	TUSC1	intergenic
**N**	oxycodone	**A**	pantoprazole	rs1048616003	16	1,23E−08	0.168	TELO2	upstream
**N**	oxycodone	**C**	furosemide	rs2291580	4	2,86E−08	0.830	CHRNA9	exonic
**N**	oxycodone	**C**	furosemide	rs181329814	7	3,34E−08	0.022	FOXK1	intergenic
**N**	tramadol	**N**	morphine	rs191619899	5	7,27E−09	0.011	GABRG2	intergenic
**N**	gabapentin	**A**	pantoprazole	rs117909688	11	1,05E−09	0.014	B3GAT1	intronic
**N**	gabapentin	**B**	acetylsal. acid	rs760791408	4	3,63E−08	0.015	RNF150	intronic

**A** Alimentary tract and metabolism **B** Blood and blood forming organs **C** Cardiovascular system **J** Anti-infectives for systemic use **M** Musculo-skeletal system **N** Nervous system.

Among the identified lead variants only two had previously been reported in any GWAS (OpenTargets [29], GWAS Catalog [33]), namely rs984113 (metoprolol + fondaparinux) which has previously been found associated with breast cancer (GCST004988) and rs76796131 (meropenem + ciprofloxacin) which was associated with smoking initiation (GCST007468) (Table S6). We tested to see if a dosage change in any particular direction (increase or decrease) was driving the association with dosage changes for any of the lead variants. We found that for 25 of the lead variants, the association was driven by either dosage increase (16 variants) or dosage decrease (9 variants) of the index drug. For an additional 21 lead variants there was an association with both dosage increase and decrease, while the remaining five variants only showed an association with dosage change and not dosage increase or decrease (Table S7).

For each drug pair phenotype, we included patients admitted with different admission diagnoses, which could influence treatment regimens and the likelihood of dosage changes. To assess whether the admission diagnosis was directly associated with the variants, we performed chi-squared testing across the 51 significant variants. For 49 of them, we found no association while two variants (rs117944645 and rs4737862) had nominally significant associations (Table S8). However, none of these were significant after correcting for multiple testing (False Discovery Rate).

Gene-based testing showed significant associations (p < 4.0E-06) among 2 of the 42 drug pair phenotypes (Table S9). Four genes were identified, three of them (*DCLRE1C*, *MEIG1*, and *OLAH*) being associated with the benzylpenicillin + furosemide phenotype while the last gene (*TMPRSS2*) was associated with the ciprofloxacin + furosemide phenotype. There was no overlap between the genes identified through significantly associated variants and those identified through gene-based testing.

### 3.3 Correlations among drug pair genetic effects

Although many of the 194 drug pair phenotypes shared index- or codrugs there was little correlation between the genetic effect sizes with the median Pearson correlation being 0.173 and 0.007 for pairs sharing the index drug and codrug, respectively. Only 59 of the in total 18,721 comparisons showed a correlation above 0.5 (min p-value = 2.02E-292) (Table S10). The majority of these, 50, came from comparisons of drug pair phenotypes with the same index drug. Among the comparisons with the highest correlations were drug pairs with the index drug clopidogrel, an antiplatelet agent, with mean correlation 0.543 (min p-value = 2.02E-292) and amiodarone, an antiarrhythmic agent, with mean correlation 0.517 (min p-value = 1.83E-144) (Figure S4). Several other index drugs also showed mean correlation above 0.5 but these were calculated from only two drug pairs each and we did not investigate them further.

### 3.4 Phenotype associations among lead SNPs

Using PheWAS we identified other phenotypes related to the 51 lead variants. PheWAS through OpenTargets identified 51 significant associations across 12 drug pair phenotypes (Table S11). We found a number of associations between variant rs4737862 (amiodarone: an antiarrhythmic agent + acetylsalicylic acid: used in pain/fever treatment) and counts of various white blood cell types as well as between variant rs6532537 (metoprolol: a beta blocker + enalapril: an ACE inhibitor prodrug) and a number of fat-related anthropometric phenotypes such (body fat percentage, trunk fat mass, etc.) The strongest association was seen between rs116091351 (pantoprazole: a proton pump inhibitor + furosemide: a diuretic) and mean platelet volume (p = 1,2E-20).

### 3.5 Genetic associations through LD, expression, and regulation

The 51 lead variants passing genome-wide significance (p < 5E-08) were located mostly in non-coding regions (25, 24, 1, and 1 variants in intronic, intergenic, upstream, and exonic regions, respectively). We explored indirect mechanisms by which the lead variants may affect drug therapy. First, we tested whether any of the lead variants were in LD with deleterious SNPs (CADD score > 12.37) in the nearest gene. None of the lead SNPs were in LD (R^2 > 0.8) with any deleterious exonic variants in the nearest gene, thus making it unlikely that any putative genetic effects are being mediated through deleterious effects from these genes. Second, we investigated whether dosage changes could be mediated through regulation of gene expression. Using eQTL catalogue annotations we found that 3 of the 51 lead variants were significantly associated with the expression of, in total, 6 genes in at least one tissue (Table S12). Among the results, rs4757645 (potassium chloride + metronidazole) was associated with increased expression of *GTF2H1,* a transcription factor subunit, in 43 separate tissues including brain, muscle, and skin tissue. Third, we queried the lead variants in RegulomeDB, a database containing experimental support for regulatory variants [34]. Eight variants received a RegulomeDB ranking of at least 1 F indicating a high probability that the variants are involved in regulation (Table S13). A few interesting observations were also made for those variants with lower RegulomeDB rankings. For example, 7 and 11 variants intersected with chromatin immunoprecipitation (CHiP) peaks in the *CTCF* and *CEBPA* genes, both of which are well-characterized transcriptional regulators. Variant rs312802 (clopidogrel + metoprolol) intersected with CHiP peaks for the *CTCF* gene from more than 400 tissue samples. Lastly, analysis of differentially expressed genes (DEGs) in FUMA across all 57 genes showed a significant (FDR < 0.05) down-regulation in salivary gland tissue (Figure S5). Separate DEG analysis of genes identified from each individual drug pair phenotype did not identify any DEGs, likely because few genes were associated with each individual drug pair phenotype.

### 3.6 Links between gene sets and disease phenotypes

We identified associations to 57 genes based on location, gene-based results, and eQTL associations (Table S14). Several of the 57 genes showed previous associations to relevant disease-related phenotypes. *SEPTIN9*, which was associated with the clopidogrel + metoprolol (an anti-platelet drug and a beta-blocker) phenotype, showed associations to blood- and cardiovascular related phenotypes such as “systolic blood pressure”, “cardiovascular disease”, and “hypertension” (OpenTargets references GCST90025968, GCST007072, and GCST90038604). Additionally, through OpenTargets’ ChEMBL annotations we found several small molecules used in pain management, including some analgesics, acting on *GABRG2* (associated with tramadol + morphine, both analgesics).

### 3.7 Gene enrichment

The 57 drug pair-associated genes could in a few cases be linked to potential drug response-affecting mechanisms. Gene set enrichment analysis (GSE) of the 57 genes together did not yield significantly associated gene sets. However, analysis of genes from several individual drug pairs did (Table S15). For example, genes LDHA and LDHC (associated with drug pair potassium chloride + metronidazole) were significantly enriched in several metabolic pathways linked to pyruvate, cysteine and methionine metabolism. Additionally, other genes associated with potassium chloride + metronidazole were enriched among GWAS hits for amyloid A serum levels.

### 3.8 Variant effects on primary care prescription-related phenotypes

Our GWASs used in-hospital data to define cases and controls. We speculated whether the identified lead variants would also affect primary care drug therapy in patients not admitted to a hospital. Using drug prescription data from DNPR covering patients not included in our GWAS analyses, we tested whether any of the 51 variants also associated with differences in drug therapy in primary care. We found no statistically significant differences in either number of prescriptions or prescribed dose for any of the variant-drug combinations (Table S16). Limiting the analysis only to periods where patients had been prescribed both drugs showed the same pattern.

## 4 Discussion

We identified 51 different lead variants associated (p < 5E-08) with dosage changes across 42 drug pair phenotypes. To our knowledge no other concurrent drug use GWAS has been reported to date. Given that only two of the 51 variants had previously been associated with a phenotype in any GWAS, this suggests that the concurrent drug use phenotype can help reveal genetic associations that may go unnoticed by studies of single-drug effects. Correlations among variant effect sizes between drug pairs were generally low, suggesting genetic backgrounds that may be more or less specific to individual drug pair combinations. This specificity may stem from the fact that many drugs act on several targets and can be metabolized by different proteins, meaning that each combination of drugs may affect a more unique set of molecular processes leading to differences in downstream effects [35]. Findings from studies of drug response in individual drugs also notice lack of overlapping loci between drugs in the same class, suggesting a high level of specificity in drug-effect pathways [36], [37].

For several of the drug pair phenotypes the associated genes could be linked to relevant disease phenotypes supporting the therapeutic relevance of those genes. For example, we found that *SEPTIN9* was associated with the clopidogrel + metoprolol drug pair phenotype. Clopidogrel is a platelet inhibitor used to decrease the risk of heart disease, while metoprolol is a beta-blocker used to treat high blood pressure. Previously *SEPTIN9* has been associated with hypertension. Our findings suggest that the association of *SEPTIN9* with those phenotypes may be mediated through drugs commonly used in treatment of those conditions [38], [39]. None of the drug pair associated genes were directly involved in processes of absorption, distribution, metabolism or excretion (ADME) of associated drugs. However, other findings provide hints at putative mechanisms for indirect drug-gene interactions. For example, *LDHA* was associated with the potassium chloride + metronidazole drug pair phenotype. Previously, antibiotic treatment (including metronidazole) in mice has shown upregulation of genes involved in anaerobic glycolysis, including *LDHA.* In vitro studies have shown that *LDH* activity potentiates metronidazole cytotoxicity and that it is reversible by *LDHA* inhibition, suggesting drug-gene mechanisms outside the commonly studied ADME processes [40], [41].While our findings do not provide direct mechanistic insight, they may provide clues for outcome-affecting genes or pathways that go beyond those identified in more disease-centered approaches.

Previous research linked our lead variants to processes of expression and regulation. Of the 57 drug pair-associated genes, six genes were linked to three of the lead variants through eQTLs. For example, we found evidence of association between rs984113 (metoprolol: a beta-blocker + fondaparinux: an anticoagulant) and *SETD9*, which is involved in DNA methylation. Previous evidence has linked *SETD9* with both coronary artery disease (GCST005196) and blood cell distribution (GCST90025988) supporting *SETD9*’s involvement in cardiovascular disease. This suggests that differentiated regulation may affect disease phenotypes through processes of altered medication patterns. The general pattern of genetic regulation affecting drug therapy has also been suggested previously, through studies of enhancers, microRNAs and other regulatory elements [40], [41]. While few have studied genetic determinants underlying multi-drug treatment there is evidence pointing to such regulatory effects from specific drug groups such as anticoagulants [42], [43].

In our analyses we found no evidence of associations between dosage changes and variants located in the “usual suspects” of pharmacogenetically important genes such as Cytochrome P450 (*CYP*) genes. Many of these genes such as *CYP2C9* and *CYP2D6* are well-described and the clinical effects of their variability likewise [44], [45]. Carrying a certain set of variants in one of these genes can classify patients into phenotypic groups such as “poor metabolizer” and “normal metabolizer”, phenotypes which have known effects on efficacy of common drugs [18]. Among the 194 drug pairs analyzed here, 99 included known metabolites of clinically actionable *CYP* genes, several with both drugs being known metabolites [46]. One explanation may be that the pathways and mechanisms important during multiple drug use may be entirely different from those which are important during monotherapy. Many *CYP* genes are involved in drug metabolism, and the absence of these genes from our results, may suggest that more complex interactions, such as changes in distribution or absorption, may be involved. Therefore, we hope that future research will expand to cover not only monotherapy, but also therapy with multiple medications, which may lead to deeper mechanistic insights.

This work shows some of the opportunities that detailed, individual-level electronic patient record data, and especially data related to drug dosing, may hold for pharmacogenomic research. Biobanks and national registries provide unique opportunities for research made possible by the ability to link various molecular level data such as genomics and proteomics to deep and longitudinal medical records [47], [48], [49], [50]. Here, we go a step further than most registry studies by looking at highly individualized in-hospital phenotypes that highlight specific changes in drug therapy. The used dosage data may be among the most accurate drug dosage measures that are systematically gathered for a large cohort and may lead to insights beyond those reached through study of lower-definition data, such as prescription records [51]. Still, this study comes with several limitations that may influence the transferability of our approach. First, dosage changes are not necessarily motivated by genetic variation. For certain drug pairs there may be clear clinical guidelines for changing the dosage, including cases in which the two drugs belong to the same drug class and/or have similar indications (e.g. tramadol and morphine). Second, differences in dosing regimens across diagnoses may affect the generalizability of our approach. Although none of the 51 significant (p < 5E-08) variants displayed evidence of being associated with the admission diagnosis, we cannot exclude that this could be the case for certain specific diagnoses when using this approach. Additionally, patients with more severe disease, such as those receiving ciprofloxacin and meropenem for example, may experience different patterns of dosage changes depending on disease progression. Given the high prevalence of multimorbidity and polypharmacy in our patient population, fine-grained analysis is challenging, partly as some treatment groups may be quite small. Third, despite the hospital drug records generally being of high quality, there may still be ambiguities in the definitions used to define patient response. For example, patients that change from a specific dose of an index drug to a “pro re nata” (i.e. use as needed) dose are here also classified as having experienced a “change”, though they may still be taking the same dose.

Future research in personalized drug therapy may focus on several opportunities. For example, investigating how the direction and magnitude of dosage changes differ among patients may complement findings from the simpler “dosage change” phenotype. Such studies may also take advantage of more advanced modelling approaches that go beyond the simple binary phenotype classifications used here and in many other genetic studies. In our study we tried to capture effects covering many drugs across many different patients. However, more targeted approaches may benefit from more specific cohorts, tailored drug dosing definitions and greater statistical power, although the number of patients will be lower. Such targeted approaches may also be used to explore more complex phenotypes, such as drug-triads and drug-disease combinations and may also study effects from known extrinsic factors such as competition for transporters and metabolic pathways which were not directly studied here. Challenges with small treatment groups may in the future be addressed with phenotype imputation, an approach which has proven successful for a number of phenotypes [52], [53]. Such methods are however, not currently able to accurately impute highly specific phenotypes without the use of other, highly correlated phenotypes, limiting their use to those cases where well-correlated proxy measures exist [53]. Recruitment and/or imputation of larger cohorts may also allow for more robust results that pass stringent multiple test thresholds, limiting inflation of false positives. Similarly, integrating machine learning approaches may complement and potentially extend current GWAS methodologies. For example, deep learning–based methods have been used to model the combined effects of multiple loci, improving phenotype prediction and uncovering novel contributors to complex traits [54]. Finally, it is important to establish evidence from diverse populations and to replicate findings. Although recent evidence has expanded our view of genetic diversity in Denmark this analysis is limited to individuals mainly of Danish / Northern European descent [55]. This may mean that our findings do not generalize to directly to other populations. We did not find a cohort with comparable, fine-grained patient-level data in which we could perform replication, and we encourage others to complement and replicate our findings by studying populations that differ from ours in their representation of ancestry, age, and morbidities.

## 5 Conclusions

In conclusion, we linked in-hospital prescription records and genetic data from Danish registries for 117,814 Danish patients and identified 51 independent loci associated with changes in drug dosage during episodes of multiple drug use. We showed that several of these loci may be coupled to processes of gene regulation and expression in genes which, to our knowledge, have not previously been linked to drug therapy outcomes. Weak correlations between the genetic effects of similar drug pair phenotypes suggest distinct genetic backgrounds among drug pairs. We hope that our approach may be useful in learning more about effects of multi-drug therapy and eventually lead to better outcomes for patients.

## Ethics approval and consent to participate

This project was approved by the Danish Data Protection Agency (P-2019–51) and the National Committee on Health Research Ethics (NVK-18038012). The study conformed to the principles of the Helsinki Declaration. Data analysis within this study was performed under the ‘Personalized medicine in oral-cardiometabolic health – the disease trajectories & genetics’ protocol (SJ-989), approved by the Danish Data Protection Agency and the Scientific Ethics Committee for the Region of Zealand (EKM-2022–03750).

## Funding

This work was supported by the 10.13039/501100009708Novo Nordisk Foundation (grants NNF170C0027594, NNF14CC0001) and the Danish Innovation Fund (grant 9090–00078A). The funding bodies had no role in the design and conduct of the study.

## CRediT authorship contribution statement

**Alexander Pil Henriksen:** Writing – review & editing, Writing – original draft, Visualization, Validation, Methodology, Investigation, Formal analysis, Data curation, Conceptualization. **Cristina Leal Rodríguez:** Methodology, Investigation, Data curation, Conceptualization. **Hannah Currant:** Writing – review & editing, Writing – original draft, Validation, Methodology**. Ioannis Louloudis:** Writing – review & editing, Validation, Methodology, Investigation, Data curation. **Jorge Hernansanz Biel:** Writing – review & editing, Data curation**. Maria Herrero-Zazo:** Writing – review & editing, Validation**. Ewan Birney:** Writing – review & editing, Validation, Supervision**. Thomas Folkmann Hansen:** Writing – review & editing, Validation, Supervision. **Gianluca Mazzoni:** Writing – review & editing, Data curation. **Amalie Dahl Haue:** Writing – review & editing, Data curation. **Henning Bundgaard:** Writing – review & editing, Data curation**. Christian Erikstrup:** Writing – review & editing, Data curation. **Khoa Manh Dinh:** Writing – review & editing, Data curation. **Liam Quinn:** Writing – review & editing, Data curation. **Mie Topholm Bruun:** Writing – review & editing, Data curation. **Henrik Hjalgrim:** Writing – review & editing, Data curation. **Erik Sørensen:** Writing – review & editing, Data curation**. Christina Mikkelsen:** Writing – review & editing, Data curation. **Michael Schwinn:** Writing – review & editing, Data curation. **Ole Birger Vestager Pedersen:** Writing – review & editing, Data curation. **Henrik Ullum:** Writing – review & editing, Data curation. **Sisse Rye Ostrowski:** Writing – review & editing, Data curation. **Karina Banasik:** Writing – review & editing, Supervision, Data curation**. Søren Brunak:** Writing – review & editing, Writing – original draft, Validation, Supervision, Project administration, Investigation, Conceptualization.

## Declaration of Competing Interest

Søren Brunak has ownerships in Hoba Therapeutics Aps, Novo Nordisk A/S, Lundbeck A/S, Eli Lilly & Co and managing board memberships in Proscion A/S. All other authors declare no competing interests.

## Data Availability

Summary statistics from the 42 drug pair phenotypes with significant variants will be made available via the GWAS Catalog. The data that support the findings of this study are available from the CHB, but restrictions apply to the availability of these data, which were used under license for the current study, and so are not publicly available. Data are however available with permission of the CHB steering committee and the Danish national scientific ethical committee.

## Footnotes

Appendix A Supplementary data associated with this article can be found in the online version at doi:10.1016/j.csbj.2025.06.042.
