# Supplementary material for: Genome-wide associations spanning 194 in-hospital drug dosage change phenotypes highlight diverse genetic backgrounds in concurrent drug therapy
Source: Comput Struct Biotechnol J. 2025 Jun 25;28:239–48. doi: 10.1016/j.csbj.2025.06.042 (PMC12268083; doi:10.1016/j.csbj.2025.06.042)

Additional file 1: Supplementary figures for Genome-wide associations spanning 194 in-hospital drug dosage change phenotypes highlight diverse genetic backgrounds in concurrent drug therapy

Alexander Pil Henriksen[1], Cristina Leal Rodríguez[2], Hannah Currant[1,3], Ioannis louloudis[1], Jorge Hernansanz Biel[1], Maria Herrero-Zazo[4], Ewan Birney[4], Thomas Folkmann Hansen[1], Gianluca Mazzoni[1], Amalie Dahl Haue[1,5], Henning Bundgaard[6,7], Christian Erikstrup[8,9], Khoa Manh Dinh[8,10], Liam Quinn[11], Mie Topholm Bruun[12], Henrik Hjalgrim[13,14], Erik Sørensen[10], Christina Mikkelsen[10], Michael Schwinn[10], Ole Birger Vestager Pedersen[7,11], Henrik Ullum[15], Sisse Rye Ostrowski[7,10], DBDS Genomic Consortium, Karina Banasik[16], Søren Brunak[1]

[1] Novo Nordisk Foundation Center for Protein Research, Faculty of Health and Medical Sciences, University of Copenhagen, Copenhagen, Denmark

[2] Copenhagen Prospective Studies on Asthma in Childhood, Copenhagen University Hospital, Gentofte, Denmark

[3] Nuffield Department for Population Health, University of Oxford, Oxford, UK

[4] European Molecular Biology Laboratory, European Bioinformatics Institute, Hinxton, UK

[5] Danish Headache Center, Department of Neurology, Copenhagen University Hospital, Rigshospitalet-Glostrup, Copenhagen, Denmark

[6] The Heart Center, Rigshospitalet, Copenhagen University Hospital, Copenhagen, Denmark

[7] Department of Clinical Medicine, Faculty of Health and Medical Sciences, University of Copenhagen, Copenhagen, Denmark

[8] Department of Clinical Immunology, Aarhus University Hospital, Aarhus, Denmark

[9] Department of Clinical Medicine, Health, Aarhus University, Aarhus, Denmark

[10] Department of Clinical Immunology, Copenhagen University Hospital - Rigshospitalet, Copenhagen, Denmark

[11] Department of Clinical Immunology, Zealand University Hospital, Køge, Denmark

[12] Department of Clinical Immunology, Odense University Hospital, Odense, Denmark

[13] Danish Cancer Society Research Center, Copenhagen, Denmark

[14] Department of Epidemiology Research, Statens Serum Institut, Copenhagen, Denmark

[15] Statens Serum Institut, Copenhagen, Denmark

[16] Department of Gynecology and Obstetrics, Copenhagen University Hospital Hvidovre, Copenhagen, Denmark

List of figures

Figure S1 – Flowchart of study process

Figure S2 – Flowchart of patient and drug pair filtering

Figure S3 – Graph showing the explained variance for each of the top 15 Principal Components

Figure S4 – Heatmap showing the pairwise beta correlations between drug pair phenotypes sharing index drug Clopidogrel or Amiodarone

Figure S5 – Bar chart from FUMA's website showing Differentially expressed genes in the 57 genes associated with drug dosage changes in this study

Figure S6 – Manhattan and QQ plots for all 42 drug pair phenotypes with at least one significant SNP

Appendix S1: Description of drug pair definition

Drug pairs were defined based on overlapping prescription periods, where two drugs were considered to be administered concurrently if the start and end dates of their prescriptions overlapped. This approach did not impose a fixed time window but relied on actual overlap in prescribed timeframes. Within each pair, the "index drug" was the medication for which a dosage adjustment occurred, while the "codrug" was the concurrently administered drug. This terminology was used to identify the direction of potential drug–drug influence on dosage modifications. A given drug could function as both index and codrug across different observations, depending on which drug underwent the dosage change. For example, if a patient received two drugs concurrently and both experienced dosage changes at different times, two drug pairs were recorded—each reflecting one drug as the index. Patients could contribute multiple drug pairs within the same admission, particularly in the context of polypharmacy; however. All 97,619 initial drug pairs were constructed using these criteria, and the final set of 194 drug pairs analyzed for dosage adjustment frequency represents a subset filtered analytically based on detectable dosage changes, with no alterations to the underlying pair construction methodology.

Supplementary Figure S1: Workflow diagram visualizing the study outline

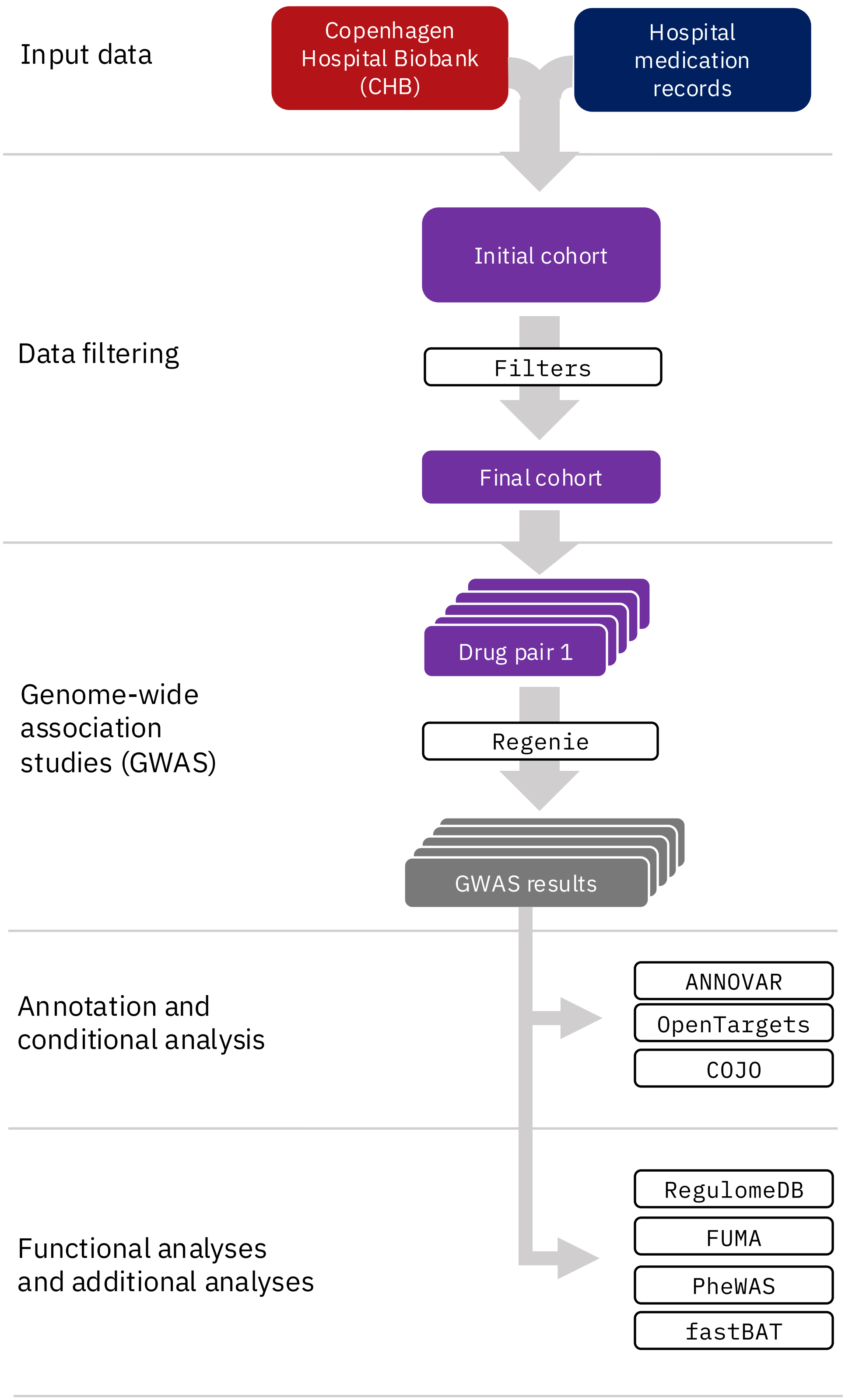

Supplementary Figure S2: Flowchart of patient and drug pair filtering.

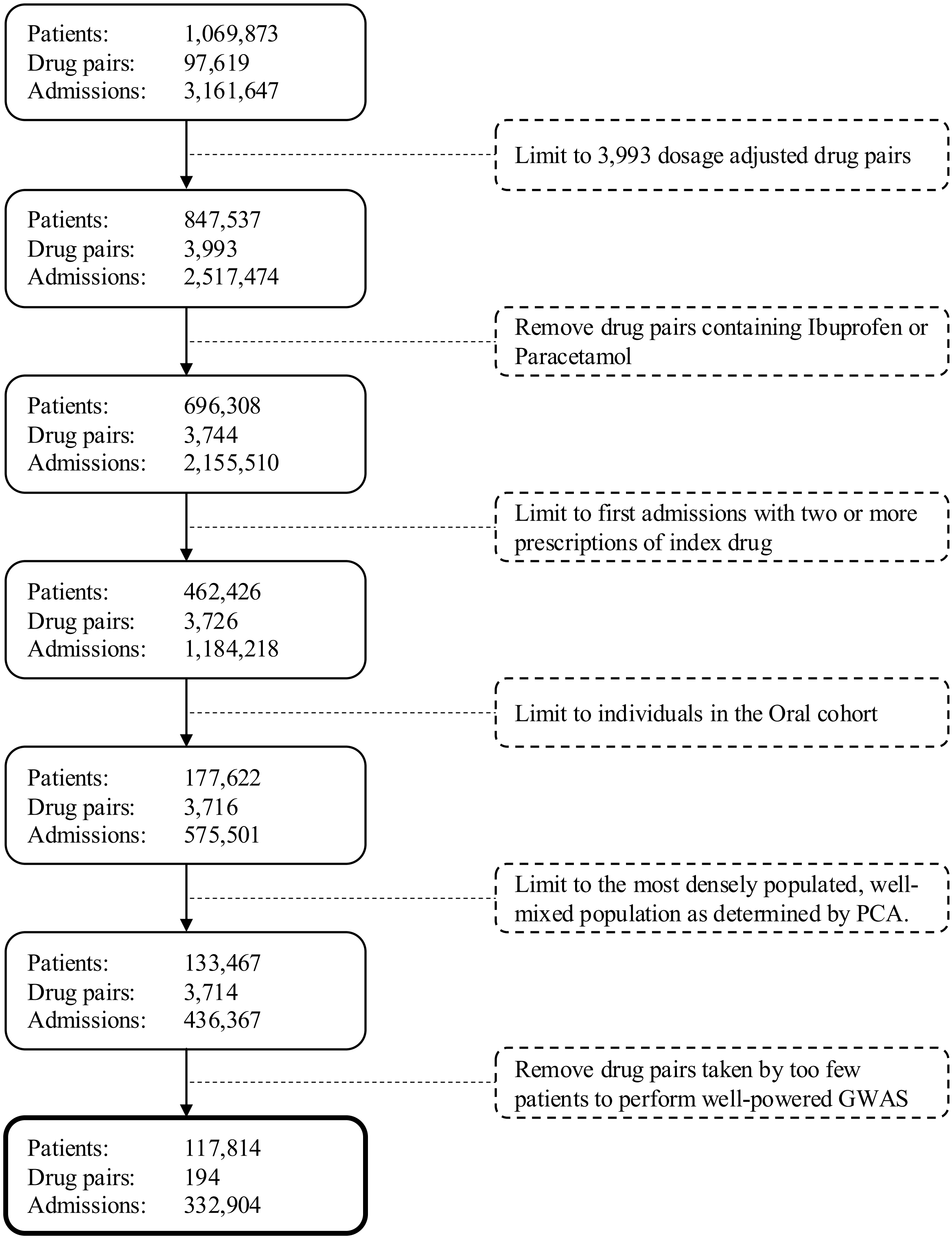

Supplementary Figure S3: Proportion of variance explained by top 15 principal components across 133,467 genotyped patients.

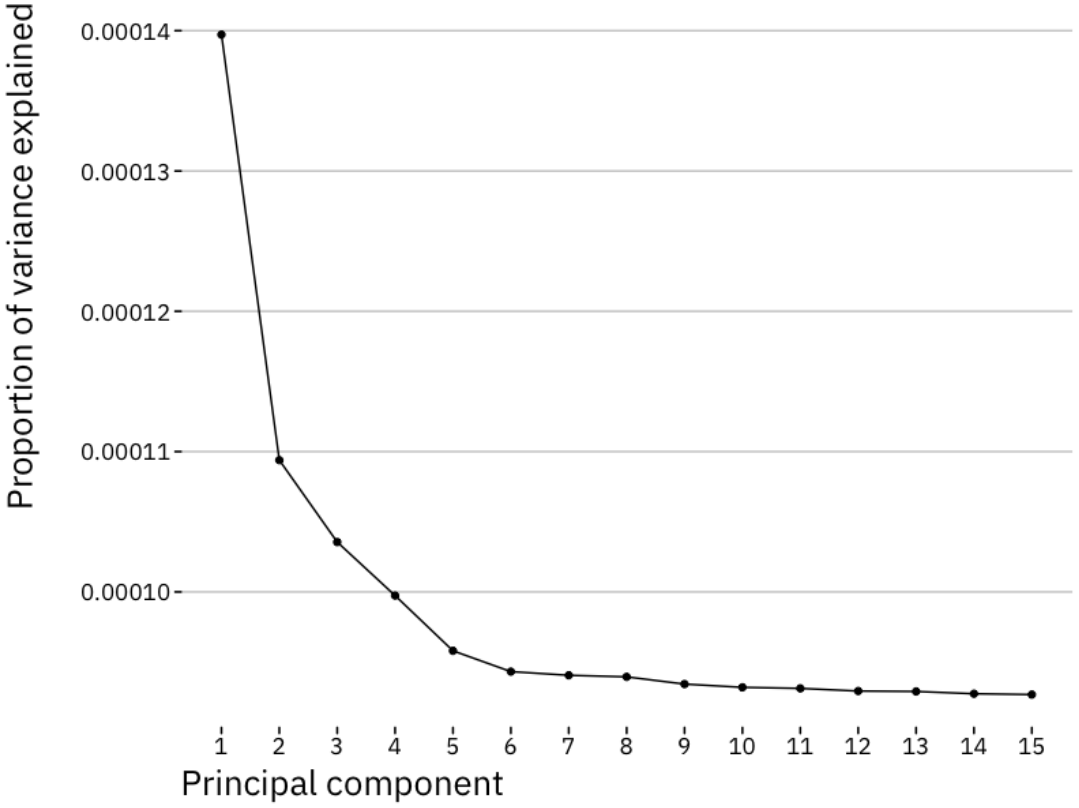

Supplementary Figure S4: Correlation of variant effect sizes among drug pair phenotypes with shared index drugs. Any variant that with a p value lower than 5e-07 in any of the GWASs was included in the analsis. Effect sizes of the resulting 974 variants are compared across drug pair phenotypes. Here, only results for clopidogrel (ATC: B01AC04) and amiodarone (ATC: C01BD01) are shown, two drugs for which the drug pair phenotypes sharing that index drug showed a high level of correlation. The x- and y-axes shown indicate the codrug in the drug pair phenotypes that are compared.

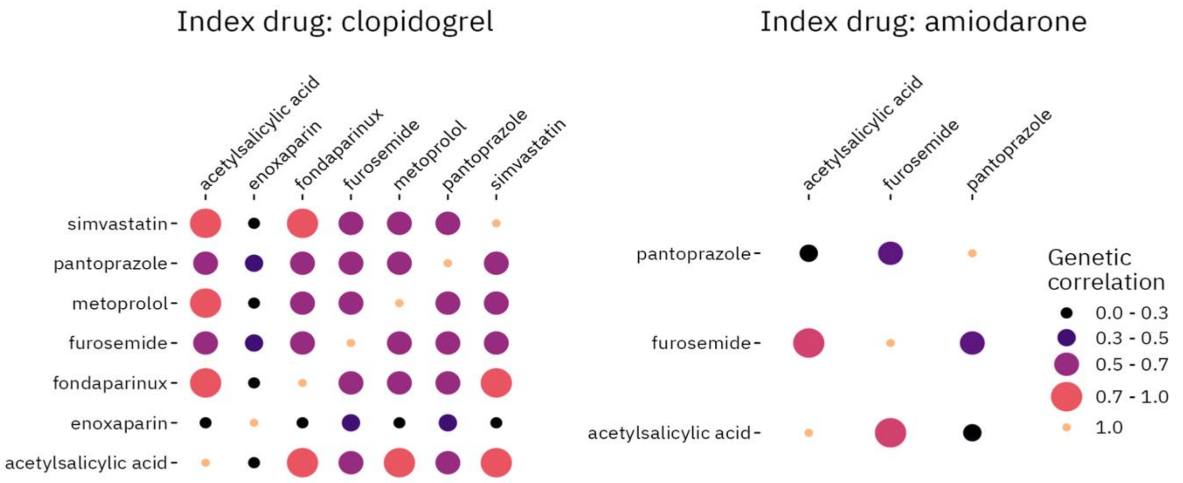

Supplementary Figure S5: Tissue specificity of differentially expressed genes (DEGs; genes showing significant changes in expression between conditions) among 59 genes associated with the 42 drug pair phenotypes where tissue specificity reflects the tendency of these DEGs to be preferentially expressed or enriched in particular tissues. Downloaded from FUMA website.

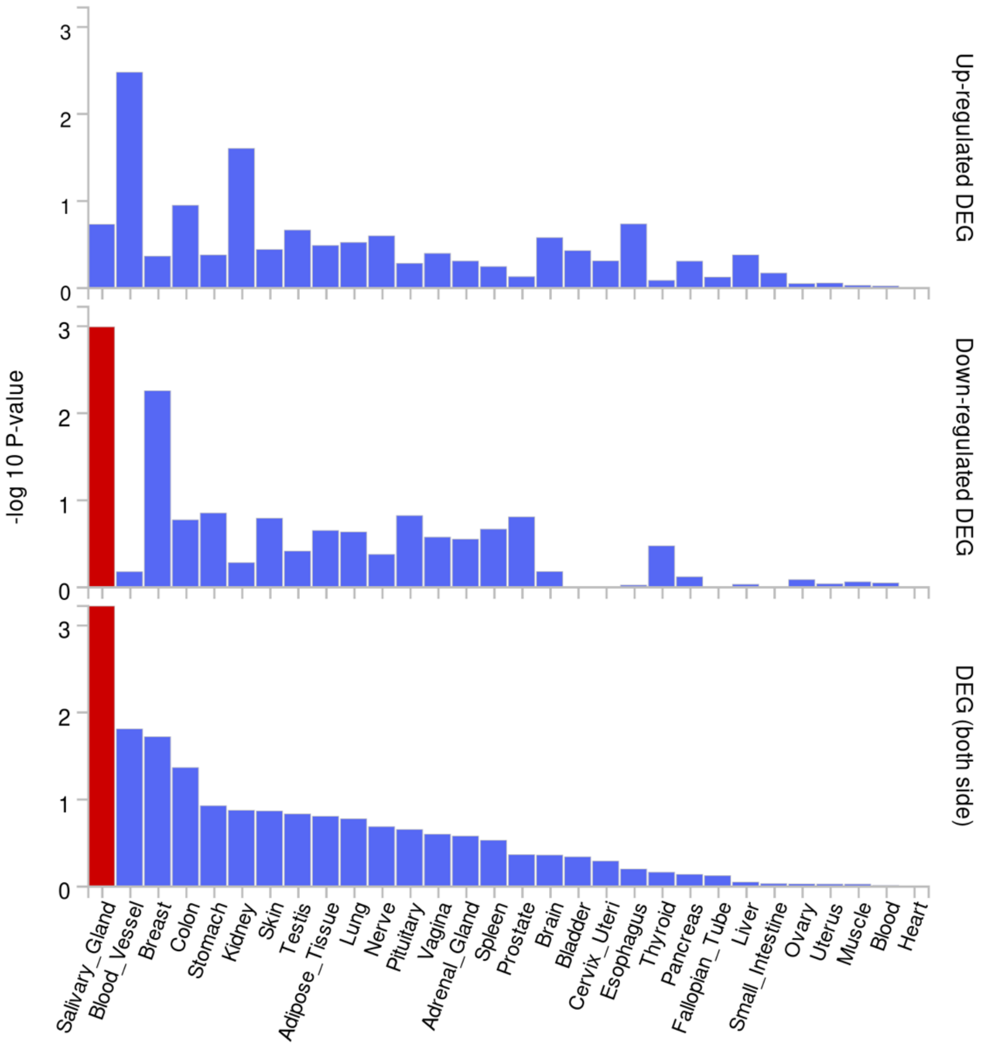

Supplementary Figure S6: Manhattan- and QQ plots of all 42 drug pair phentoypes with at least one genome-wide significant

A02AA04_N02AA01: magnesium hydroxide + morphine

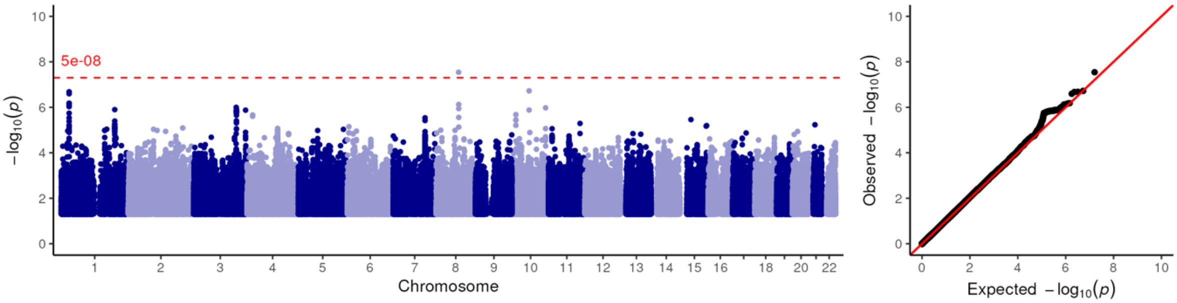

A02BC02_A03FA01: pantoprazole + metoclopramide

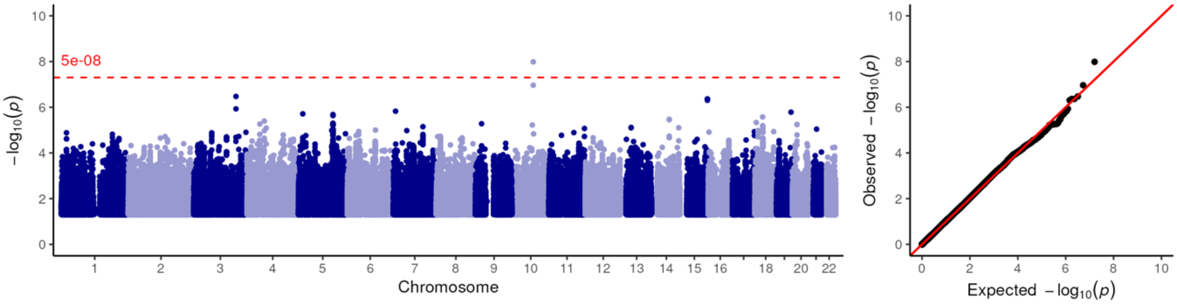

A02BC02_C03CA01: pantoprazole + furosemide

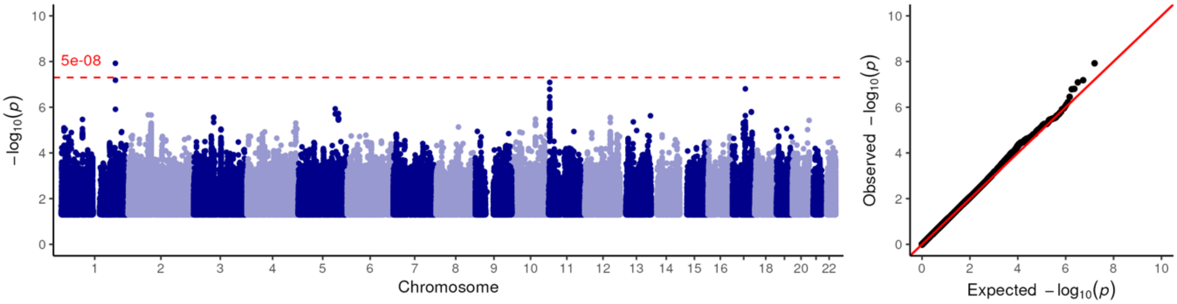

A02BC02_J01MA02: pantoprazole + ciprofloxacin

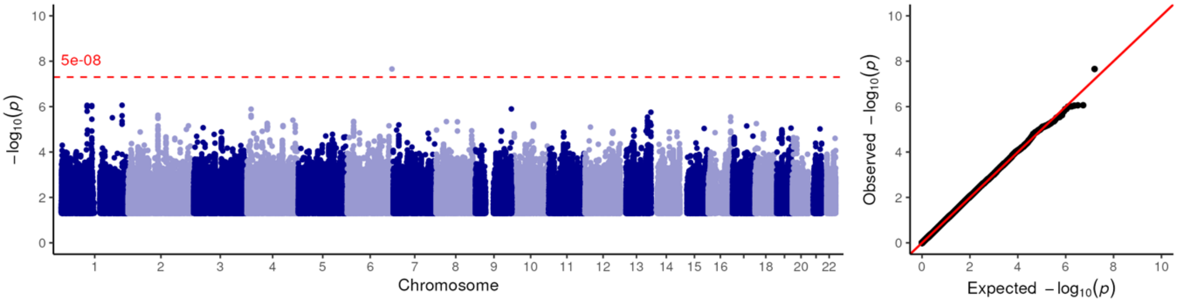

Supplementary Figure 5: Manhattan- and QQ plots of all 42 drug pair phentoypes with at least one genome-wide significant

A06AD11_A06AB08: lactulose + sodium picosulfate

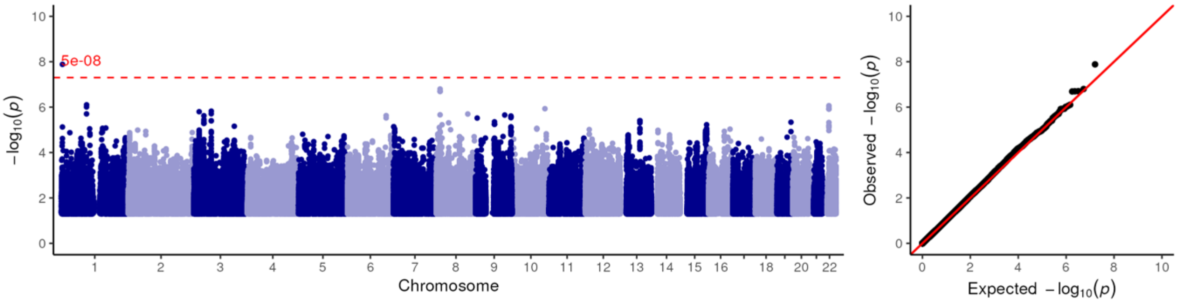

A06AD11_C03CA01: lactulose + furosemide

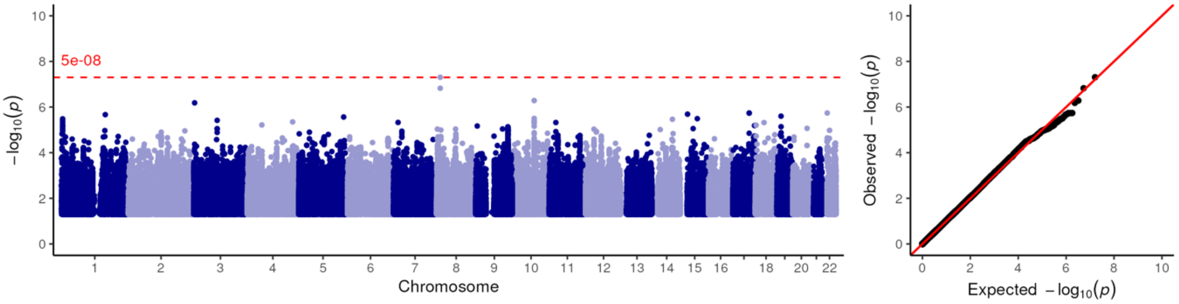

A11DA01_J01XD01: thiamine (vit B1) + metronidazole

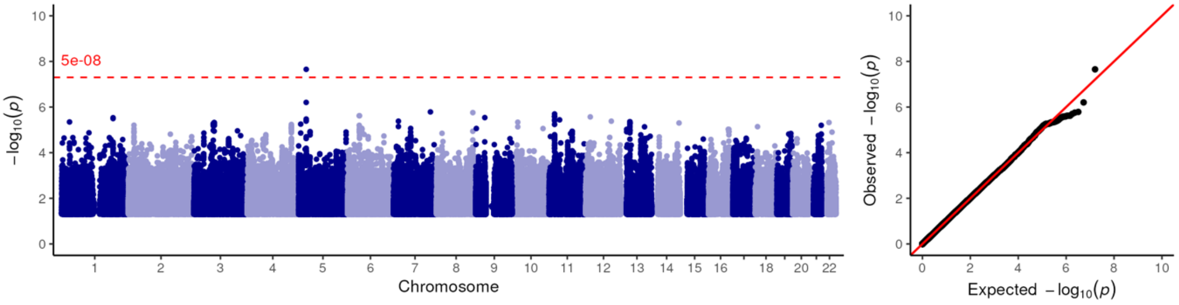

A11DA01_N05BA02: thiamine (vit B1) + chlordiazepoxide

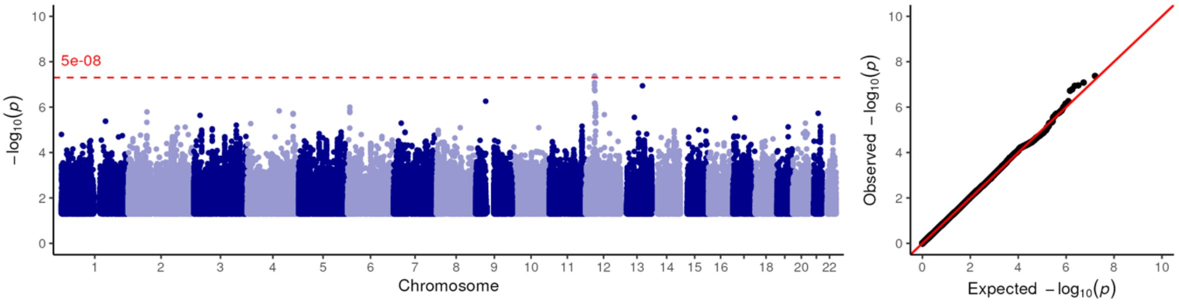

Supplementary Figure 5: Manhattan- and QQ plots of all 42 drug pair phentoypes with at least one genome-wide significant

A12BA01_A12CC30: potassium chloride + magnesium (different salts in combination)

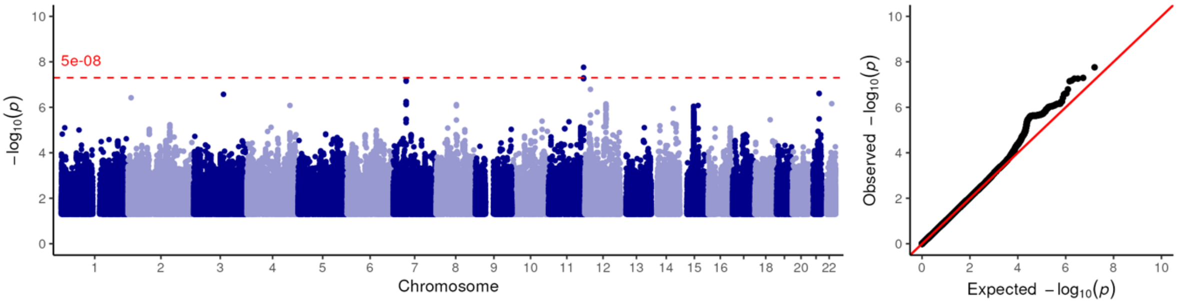

A12BA01_C03CA01: potassium chloride + furosemide

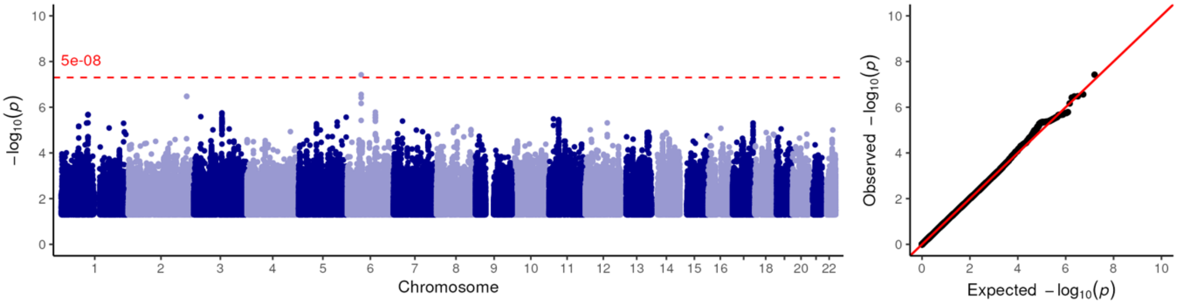

A12BA01_J01MA02: potassium chloride + ciprofloxacin

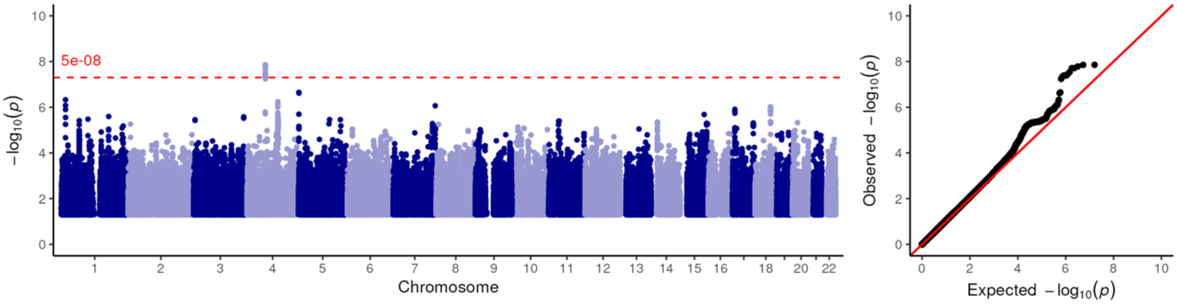

A12BA01_J01XD01: potassium chloride + metronidazole

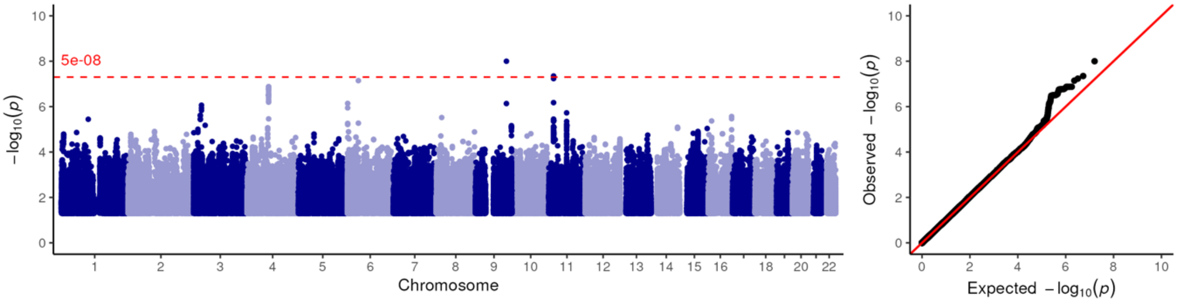

Supplementary Figure 5: Manhattan- and QQ plots of all 42 drug pair phentoypes with at least one genome-wide significant

A12BA01_N05BA02: potassium chloride + chlordiazepoxide

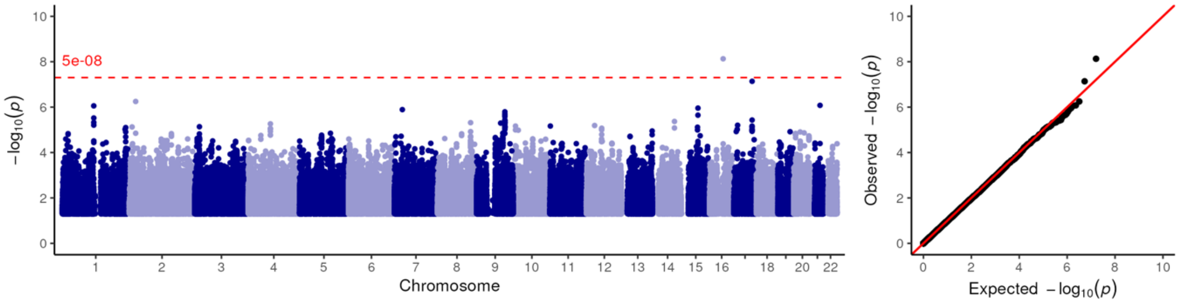

B01AA03_C03CA01: warfarin + furosemide

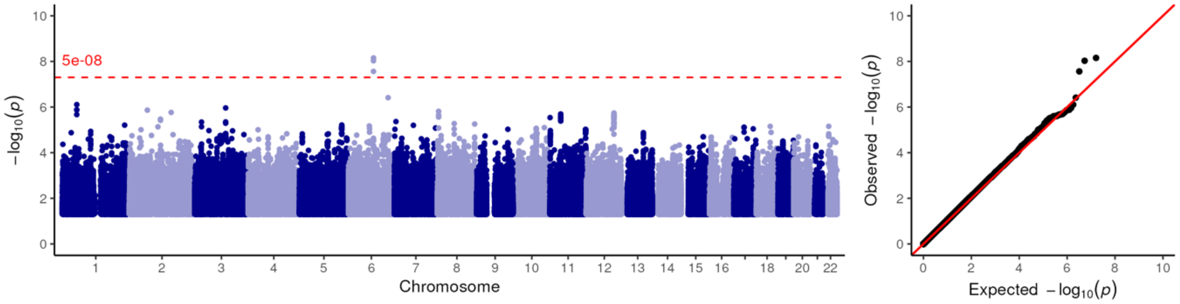

B01AB10_A12BA01: tinzaparin + potassium chloride

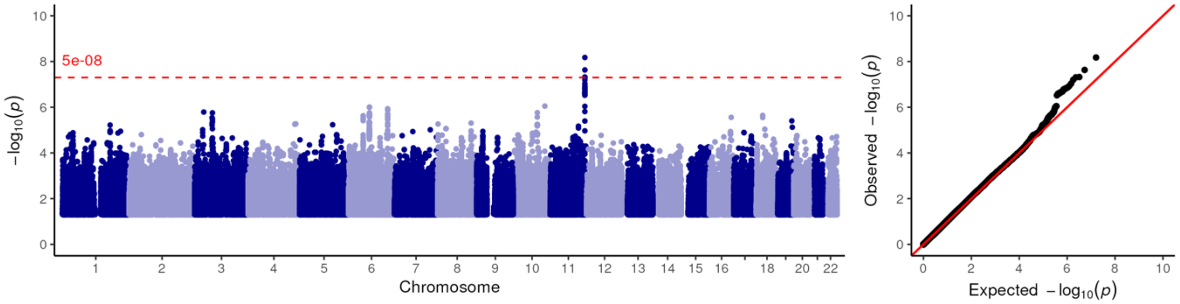

B01AC04_B01AC06: clopidogrel + acetylsalicylic acid

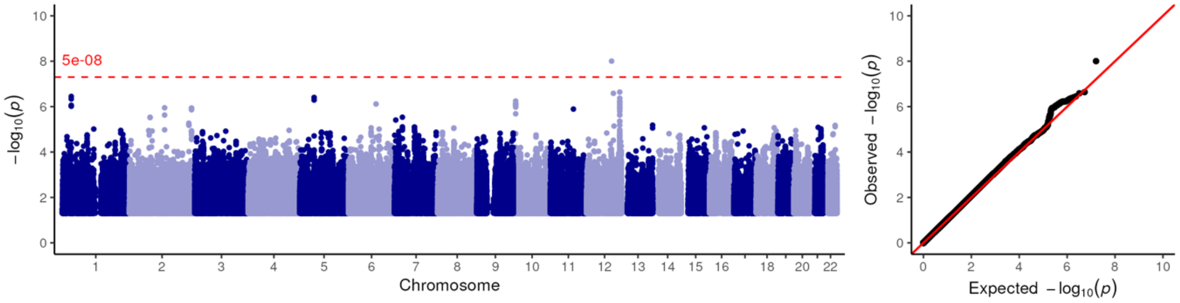

Supplementary Figure 5: Manhattan- and QQ plots of all 42 drug pair phentoypes with at least one genome-wide significant

B01AC04_C07AB02: clopidogrel + metoprolol

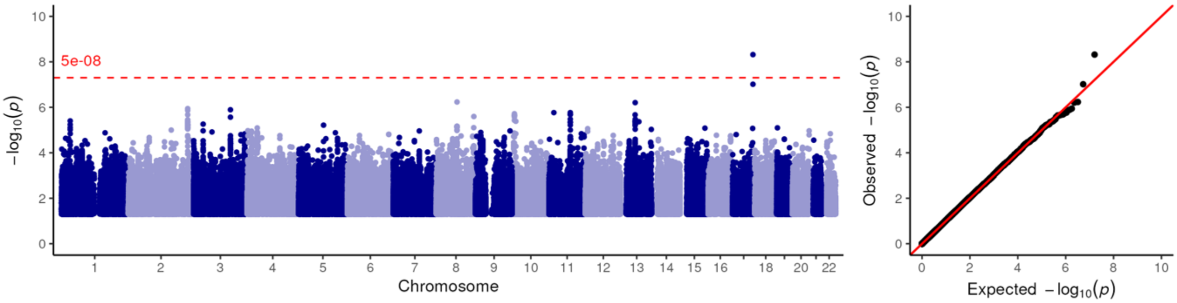

B01AC04_C10AA01: clopidogrel + simvastatin

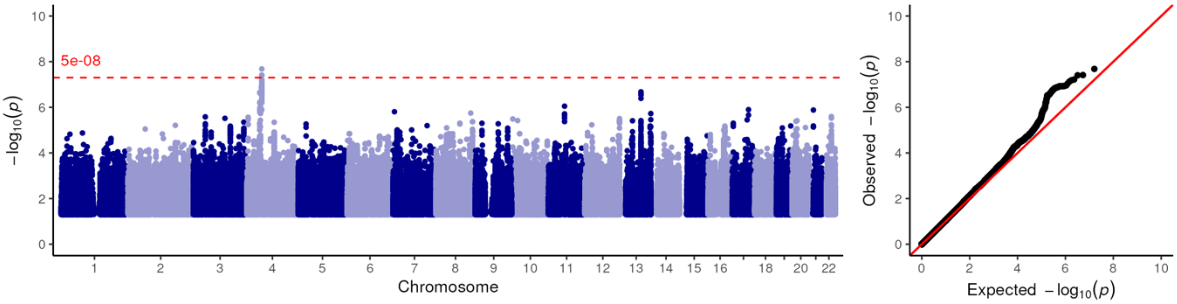

B01AC06_C07AB02: acetylsalicylic acid + metoprolol

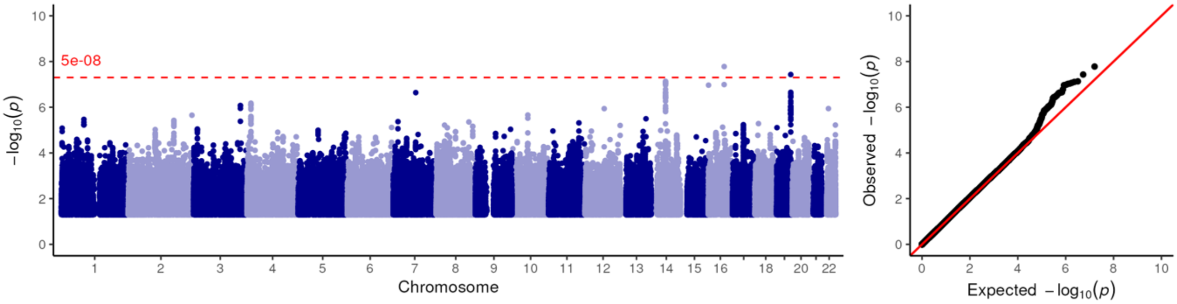

C01AA05_A12BA01: digoxin + potassium chloride

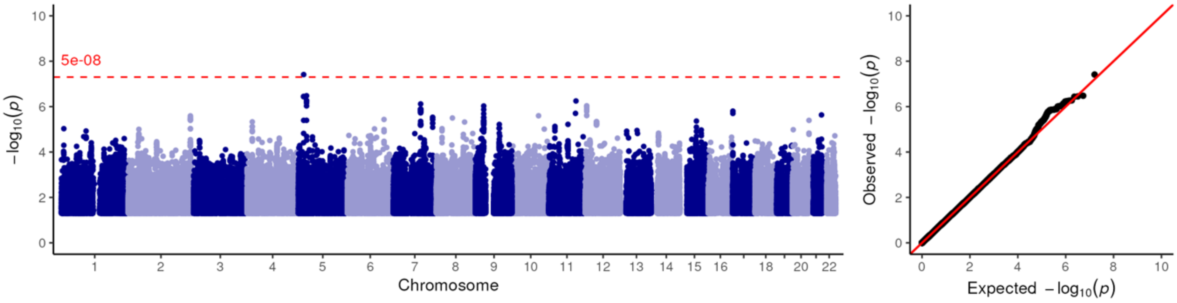

Supplementary Figure 5: Manhattan- and QQ plots of all 42 drug pair phentoypes with at least one genome-wide significant

C01BD01_A02BC02: amiodarone + pantoprazole

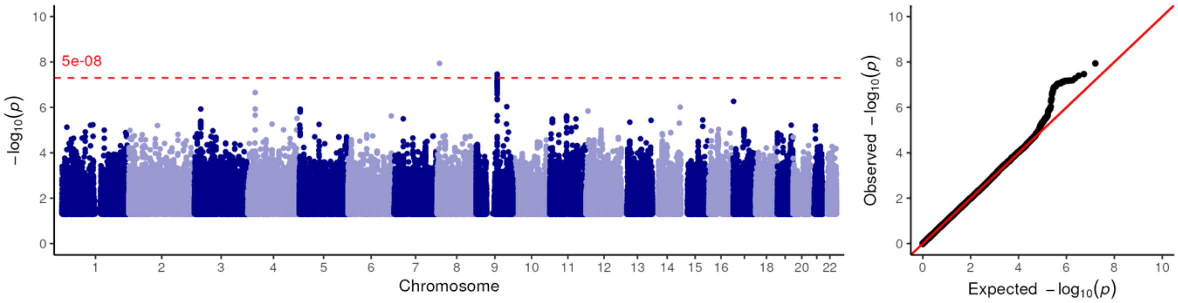

C01BD01_B01AC06: amiodarone + acetylsalicylic acid

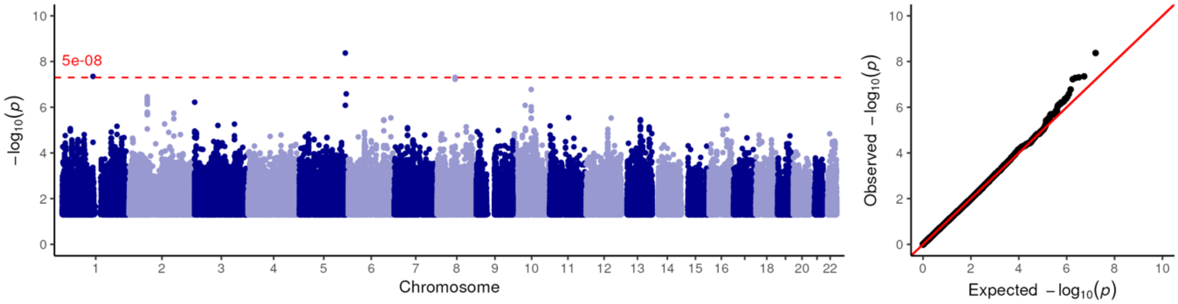

C03CA01_C03DA01: furosemide + spironolactone

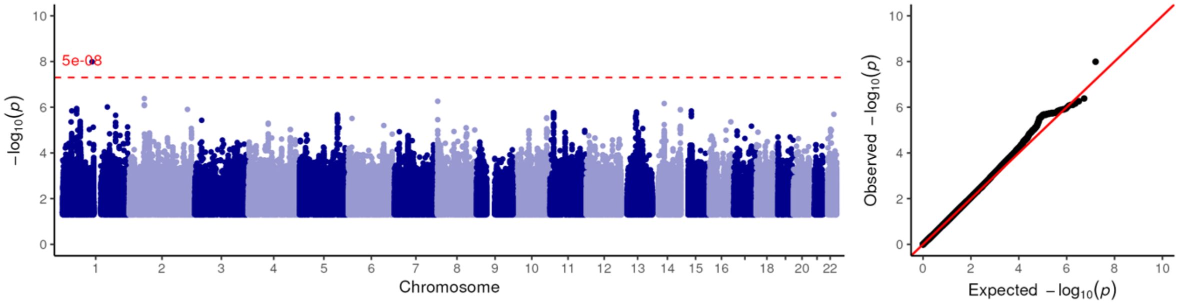

C07AB02_B01AC24: metoprolol + ticagrelor

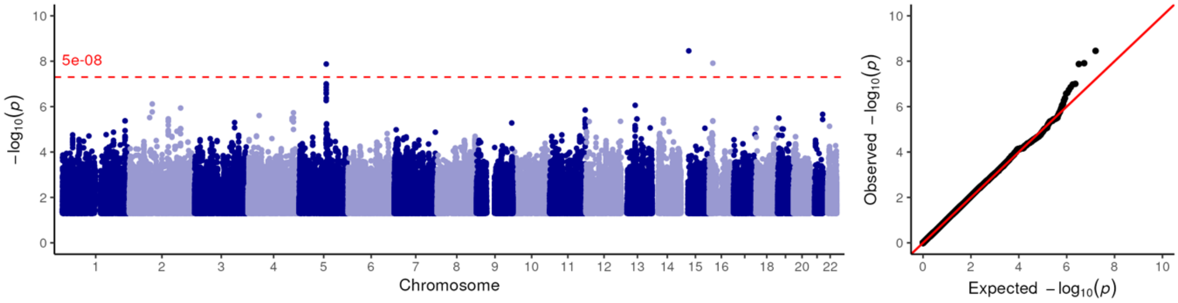

Supplementary Figure 5: Manhattan- and QQ plots of all 42 drug pair phentoypes with at least one genome-wide significant

C07AB02_B01AX05: metoprolol + fondaparinux

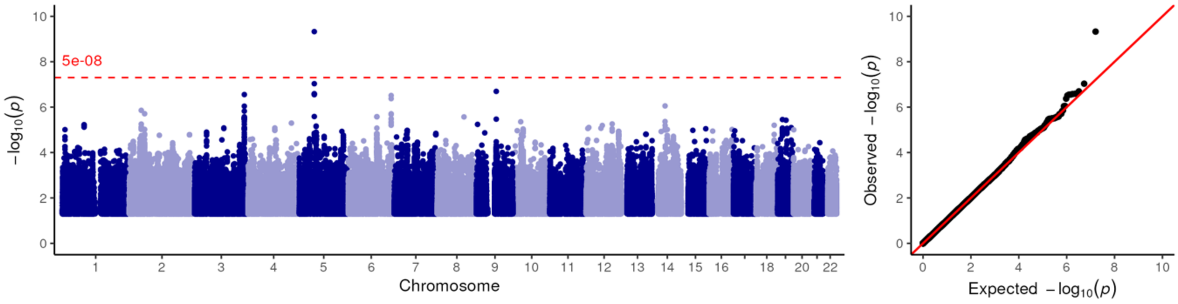

C07AB02_C09AA02: metoprolol + enalapril

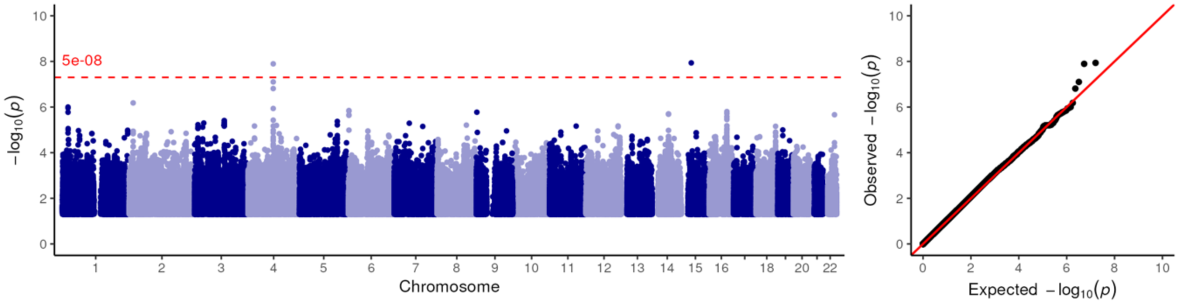

J01CE01_C03CA01: benzylpenicillin + furosemide

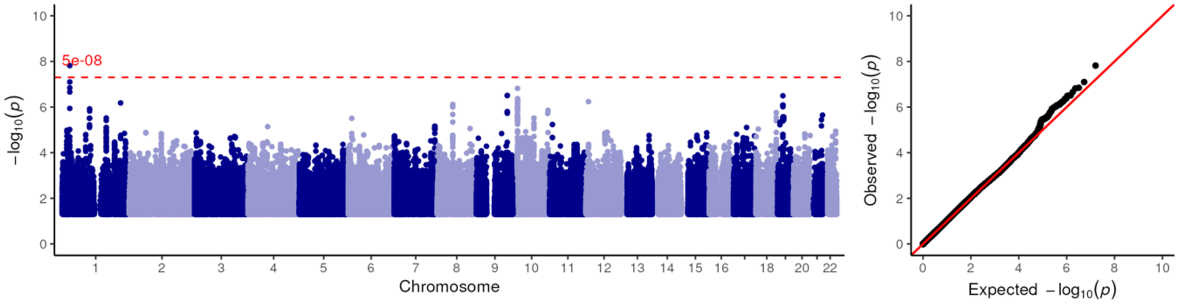

J01CF01_C03CA01: dicloxacillin + furosemide

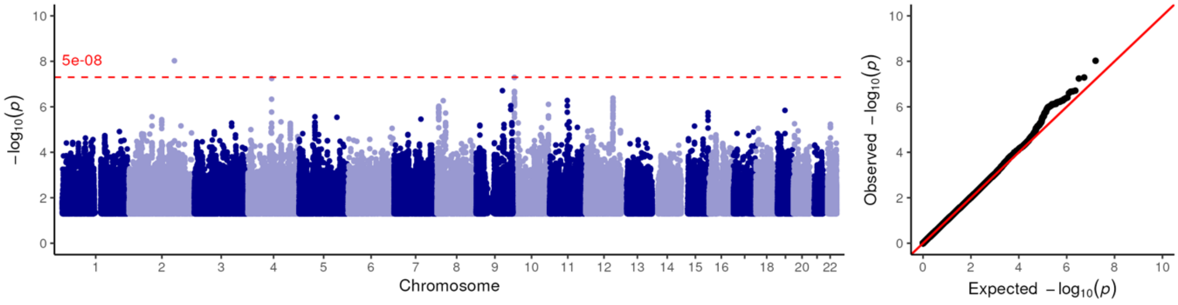

Supplementary Figure 5: Manhattan- and QQ plots of all 42 drug pair phentoypes with at least one genome-wide significant

J01CF01_N02AA05: dicloxacillin + oxycodone

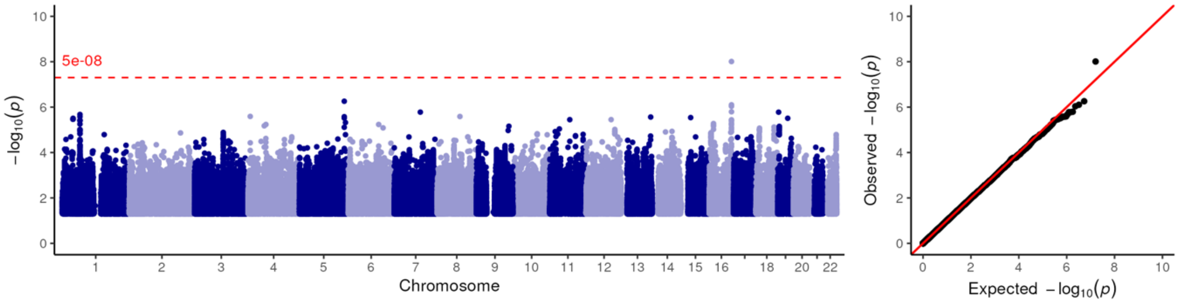

J01CF01_N03AX12: dicloxacillin + gabapentin

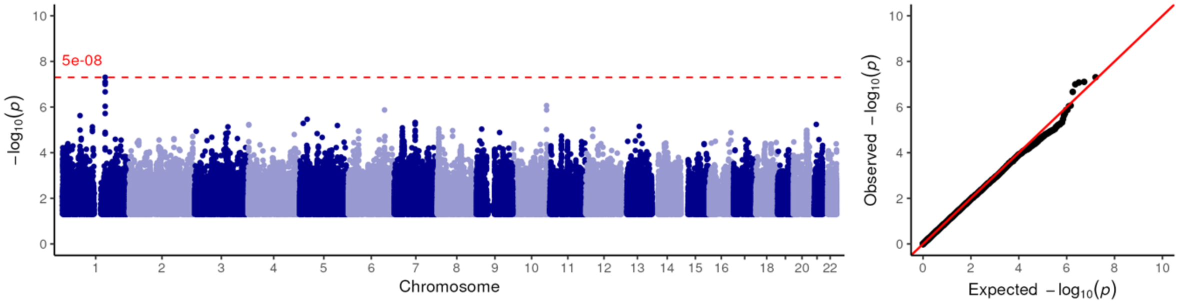

J01DC02_M03BB03: cefuroxime + chlorzoxazone

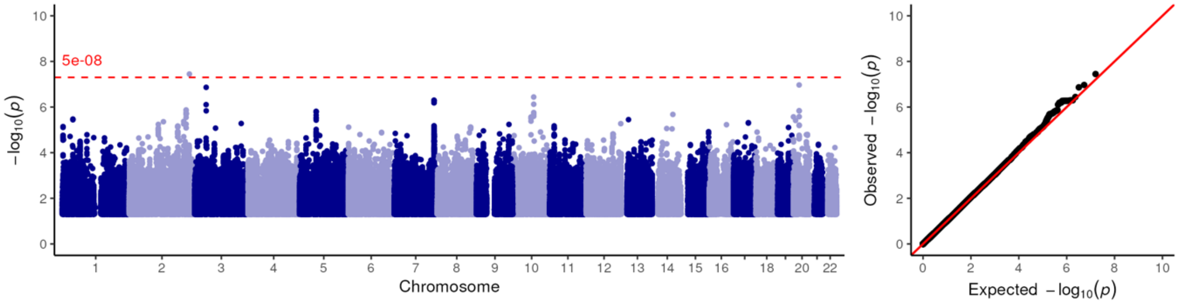

J01DC02_N01BB01: cefuroxime + bupivacaine

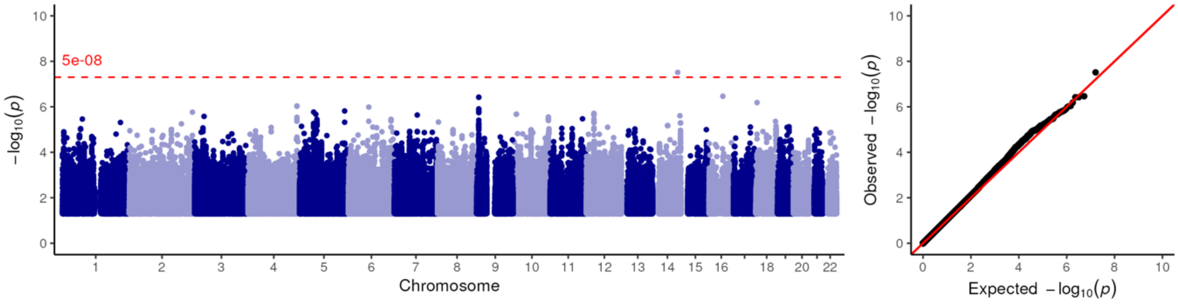

Supplementary Figure 5: Manhattan- and QQ plots of all 42 drug pair phentoypes with at least one genome-wide significant

J01DH02_B01AC06: meropenem + acetylsalicylic acid

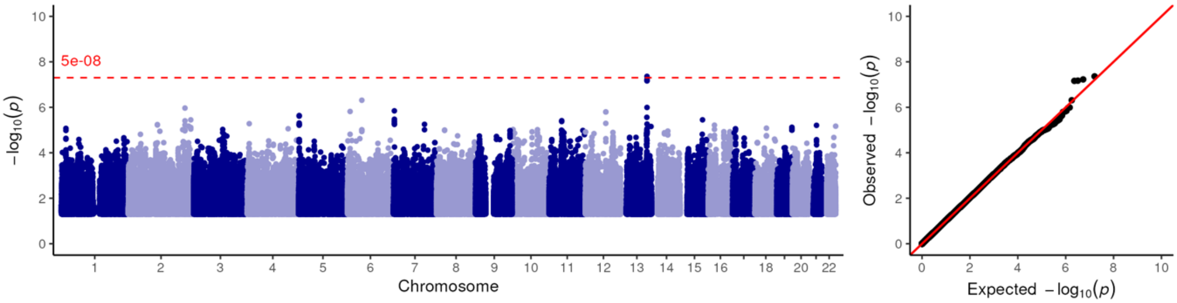

J01DH02_J01MA02: meropenem + ciprofloxacin

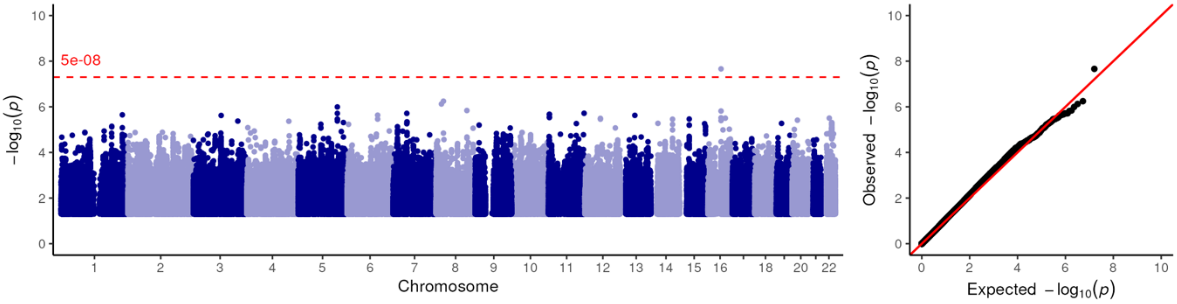

J01MA02_A11DA01: ciprofloxacin + thiamine (vit B1)

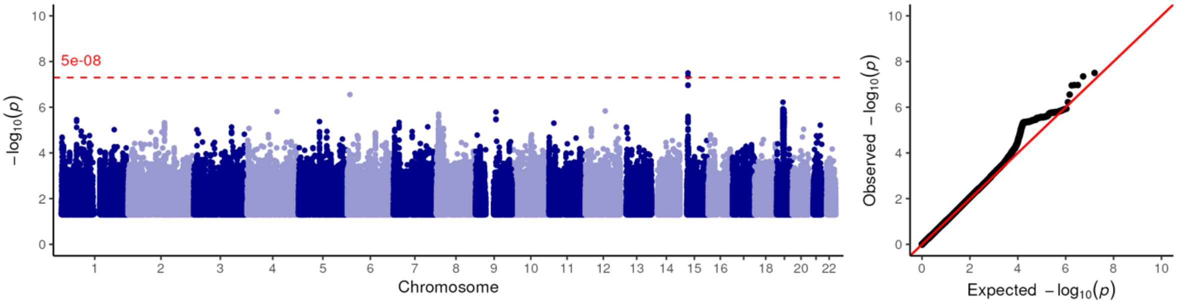

J01MA02_C03CA01: ciprofloxacin + furosemide

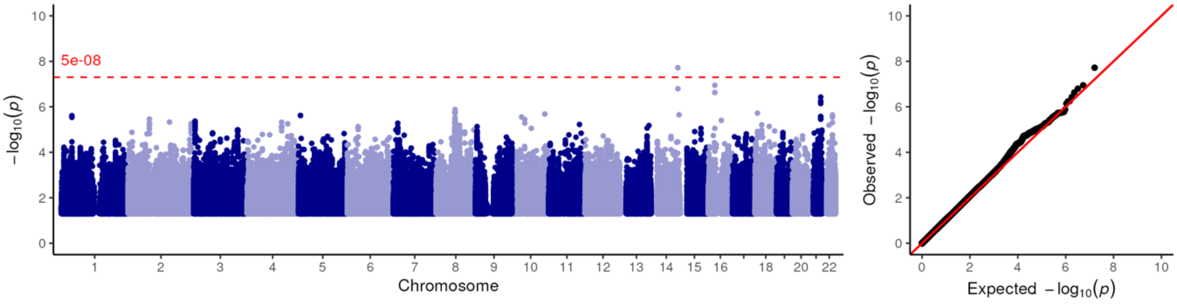

Supplementary Figure 5: Manhattan- and QQ plots of all 42 drug pair phentoypes with at least one genome-wide significant

J01MA02_J01XD01: ciprofloxacin + metronidazole

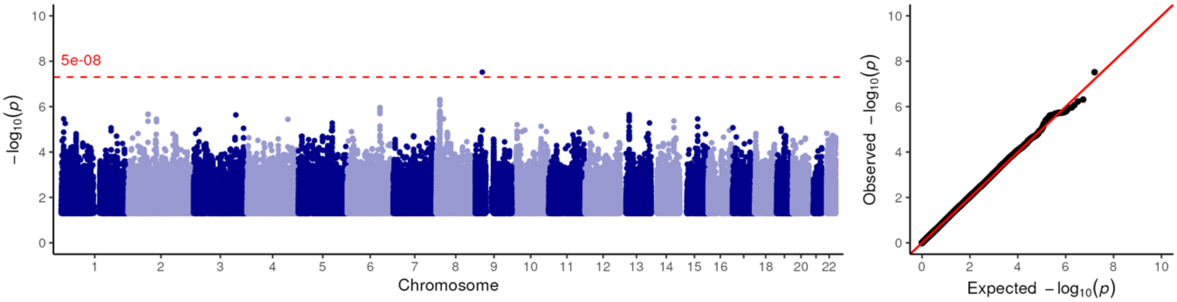

N02AA05_A02BC02: oxycodone + pantoprazole

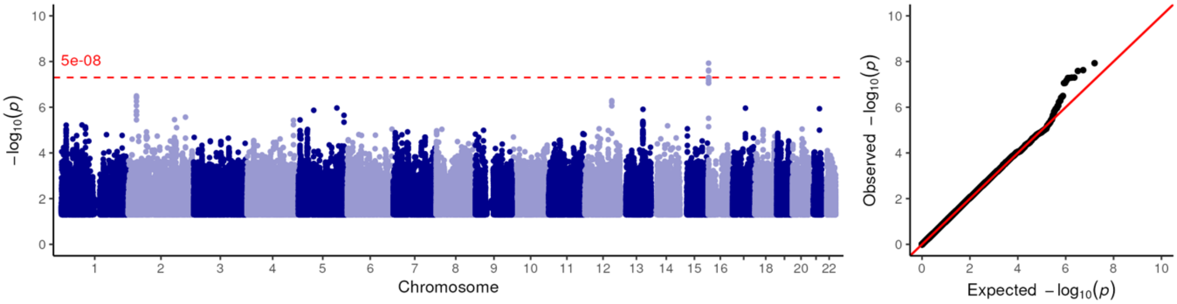

N02AA05_C03CA01: oxycodone + furosemide

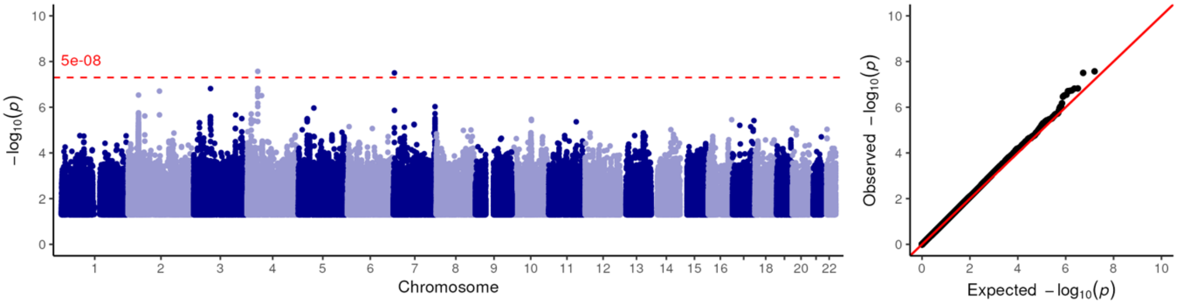

N02AX02_N02AA01: tramadol + morphine

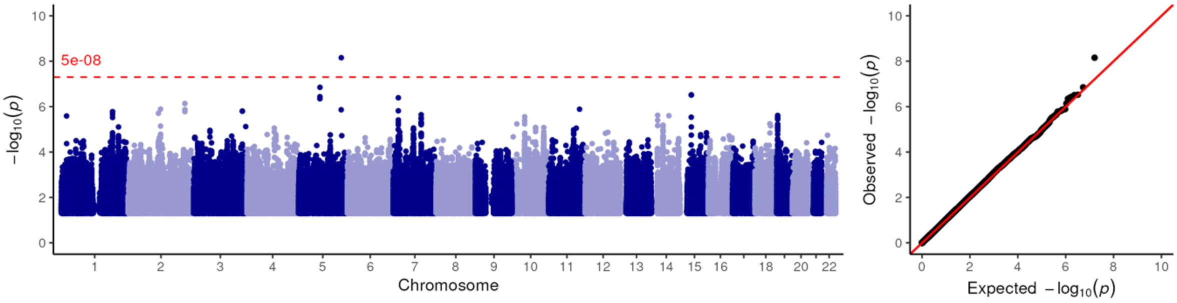

Supplementary Figure 5: Manhattan- and QQ plots of all 42 drug pair phentoypes with at least one genome-wide significant

N03AX12_A02BC02: gabapentin + pantoprazole

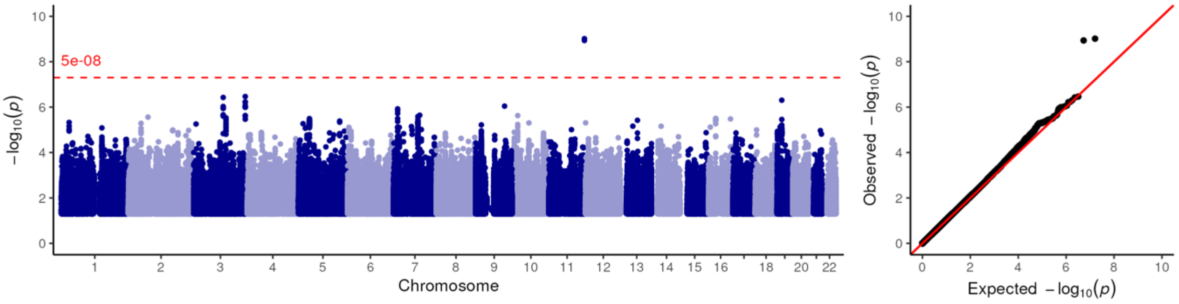

N03AX12_B01AC06: gabapentin + acetylsalicylic acid

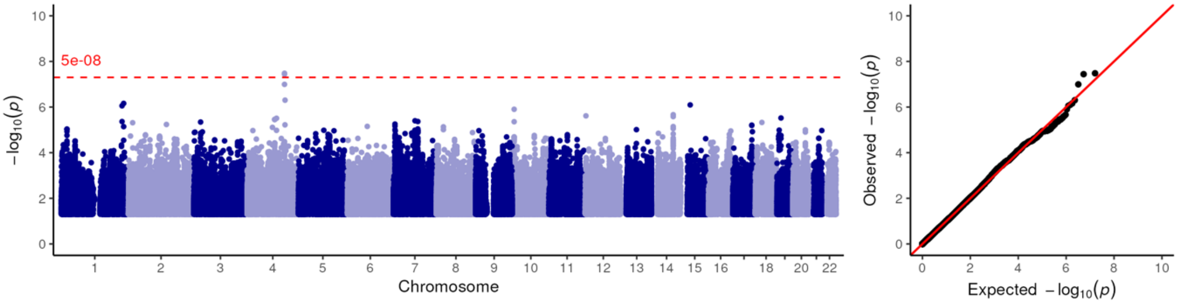

Supplement: Supplementary file 1 — Supplementary material [file mmc1.pdf]
